# Plasma Cell Differentiation Pathways in Systemic Lupus Erythematosus

**DOI:** 10.3389/fimmu.2018.00427

**Published:** 2018-03-05

**Authors:** Susan Malkiel, Ashley N. Barlev, Yemil Atisha-Fregoso, Jolien Suurmond, Betty Diamond

**Affiliations:** ^1^Center of Autoimmune Musculoskeletal and Hematopoietic Diseases, The Feinstein Institute for Medical Research, Northwell Health, Manhasset, NY, United States; ^2^Donald and Barbara Zucker School of Medicine at Hofstra/Northwell, Hempstead, NY, United States; ^3^Tecnologico de Monterrey, Monterrey, Mexico

**Keywords:** systemic lupus erythematosus, autoantibodies, B cells, plasma cells, tolerance

## Abstract

Plasma cells (PCs) are responsible for the production of protective antibodies against infectious agents but they also produce pathogenic antibodies in autoimmune diseases, such as systemic lupus erythematosus (SLE). Traditionally, high affinity IgG autoantibodies are thought to arise through germinal center (GC) responses. However, class switching and somatic hypermutation can occur in extrafollicular (EF) locations, and this pathway has also been implicated in SLE. The pathway from which PCs originate may determine several characteristics, such as PC lifespan and sensitivity to therapeutics. Although both GC and EF responses have been implicated in SLE, we hypothesize that one of these pathways dominates in each individual patient and genetic risk factors may drive this predominance. While it will be important to distinguish polymorphisms that contribute to a GC-driven or EF B cell response to develop targeted treatments, the challenge will be not only to identify the differentiation pathway but the molecular mechanisms involved. In B cells, this task is complicated by the cross-talk between the B cell receptor, toll-like receptors (TLR), and cytokine signaling molecules, which contribute to both GC and EF responses. While risk variants that affect the function of dendritic cells and T follicular helper cells are likely to primarily influence GC responses, it will be important to discover whether some risk variants in the interferon and TLR pathways preferentially influence EF responses. Identifying the pathways of autoreactive PC differentiation in SLE may help us to understand patient heterogeneity and thereby guide precision therapy.

## Introduction

Systemic lupus erythematosus (SLE) is a systemic autoimmune disease characterized by the production of pathogenic autoantibodies that target a variety of nuclear self-antigens, some of which cross-react with tissue antigens. These autoantibodies cause tissue inflammation and lead to organ damage in the kidneys, skin, and more. Presence of IgG ANA is a diagnostic feature for SLE and other systemic autoimmune diseases, and these antibodies have an important role in disease pathogenesis (1, 2). In contrast, IgM ANA are considered to be protective against autoimmunity. They can be present in healthy individuals and assist in the non-inflammatory clearance of cellular debris and inhibit responses induced by IgG ANA (3–5). Other isotypes include IgA and IgE, but the pathogenicity of these isotypes has been less well studied.

Antibodies are secreted by plasma cells (PCs), which arise as a terminal differentiation step from B cells. Most of our knowledge of immune tolerance to nuclear antigens, and the break of tolerance in SLE patients, is derived from studies with B cell receptor (BCR)-transgenic mice and single cell studies in humans, where self-reactivity is usually censored in developing B cells prior to their achieving immunocompetence (6, 7). Autoreactive cells that escape these mechanisms often become anergic (8–10), a process that mitigates against these cells giving rise to high affinity IgG autoantibody-producing PCs.

Plasma cells can arise through two pathways: through activation of B cells and direct differentiation in extrafollicular (EF) foci or through a germinal center (GC) response. Although traditionally pathogenic high affinity autoantibodies have been associated with the GC response, recent insights have implicated the EF pathway in SLE as well. We hypothesize that both pathways can contribute to production of SLE autoantibodies. Understanding the regulation of each pathway and how genetic risk alleles may preferentially target one or the other of these pathways will be the focus of this review.

Different subtypes of PCs have been described, including plasmablasts (PB), pre-PC, early PC, short-lived PC, and long-lived PC. These terms are sometimes used interchangeably or not clearly defined. The confusion in part derives from the original paradigm that the EF pathway only results in short-lived proliferating PBs, whereas the GC pathway was thought to result only in long-lived quiescent PCs (11). However, lifespan and proliferation can operate independently from each other, such that there are short-lived PCs which are not proliferating, and long-lived PCs from GC origin can proliferate prior to becoming quiescent (12–14). In addition, PC differentiation is a continuum where expression of canonical B cell markers [B220, CD19, major histocompatibility complex (MHC) class II] is gradually lost and PC markers (such as Blimp-1, CD138, secreted Ig) are gradually upregulated (14). It is therefore difficult to define specific PC subsets based on the expression of these markers. Here, we define PCs as antibody secreting cells and we will only mention specific PC subsets if these have been clearly verified. Definitions used in this review are PBs, if proliferation has been verified; short-lived plasma cells, if a short lifespan of <7 days has been shown; or long-lived PC, if a long lifespan of >28 days has been demonstrated.

## T-Independent B Cell Activation and PC Differentiation

The B cell lineage consists of several subsets and cells diverge early in development. Each of these naive cell subsets can give rise to PCs, but they each preferentially respond to specific types of antigen. Antigens can activate B cells in a T-independent or T-dependent manner. T-independent responses do not require cognate T cell help. T-independent activation therefore leads to plasma cell differentiation in the absence of GCs. There are two types of T-independent antigens that can induce activation of B cells; TI-1 antigens can activate B cells through coengagement of Toll-like receptors (TLR), such as LPS or other bacterial polysaccharides, whereas TI-2 antigens lead to extensive crosslinking of the BCR, such as polymeric protein antigens or repeated structural motifs (15). In TI-2 responses, competition for antigen enhances the activation and expansion of high-affinity cells, while antigen affinity is less important in TI-1 responses (16).

Although TI-1 and TI-2 antigens have been considered to induce T cell-independent responses, it is now clear that this distinction is not absolute: TI-2 and possibly TI-1 antigens can induce a transient GC (17), and the TI-2 serum antibody responses, in particular IgG, can still be T cell-dependent, even if the antigen cannot directly trigger T cells through MHC class II (18). It has therefore been proposed that the characteristics of the antigen is not the leading determinant of the response, but rather the B cell subset and the ancillary cell types involved determine the nature of the response (19).

In addition to the strength of the initial stimulus through the BCR and cognate and non-cognate T cell interactions, B cell activation is also modified by the presence of other potent stimuli. Pattern recognition receptors, such as TLRs, interact with damage-associated molecular patterns or highly conserved microbial structures present in bacteria or virus. Included among these are both dsDNA (CpG enriched) and RNA. Many TLRs signal through MyD88, and MyD88-deficient mice have diminished antibody responses, both early and late after immunization (20–23). Simultaneous engagement of the BCR and TLRs has a synergistic effect on signaling and subsequent B cell activation (24).

Cytokines, such as type I interferon (IFN), IL-6, and BAFF, can activate B cells and enhance both T-independent and T-dependent activation (25–27). BAFF has three recognized receptors, and one of them, TACI, signals through MyD88 (28), the same adaptor used by TLR7 and TLR9. Antigen-presenting cells, such as dendritic cell (DC) and macrophages, induce CD40-independent PC differentiation through secretion of cytokines such as BAFF and APRIL (26).

Two processes that alter the antibody response are somatic hypermutation (SHM) and class switch recombination (CSR), both of which are mediated by the enzyme activation-induced cytidine deaminase (AID) (29). These processes can change antigen recognition by the BCR (SHM) or change the isotype that is expressed (CSR), and are usually associated with GC responses (discussed below). Although T-independent responses are usually associated with the IgM isotype, CSR can occur in certain infections and does not require cognate T–B interactions (30). CSR can be driven by MyD88 signaling or cytokines such as BAFF, APRIL, IFN-gamma, and IL-21 (20, 23, 31, 32).

### B-1 Cells

**Figure 1:**
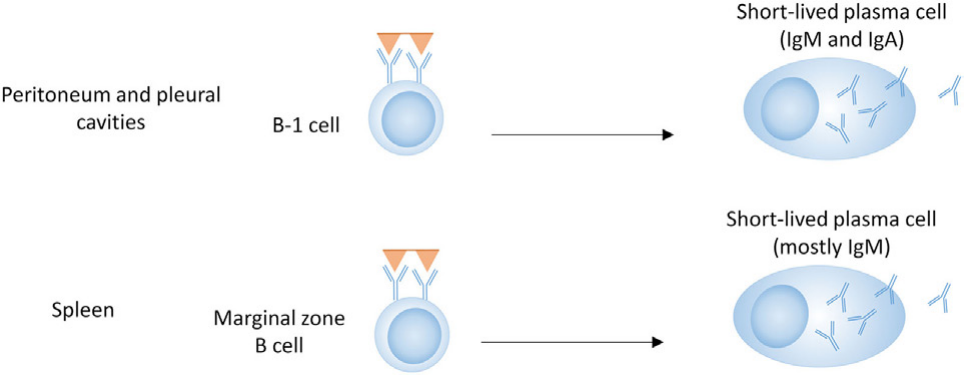
B cell subsets in T-independent plasma cell development. B-1 cells in the peritoneal and pleural cavities can produce antibodies of the IgM and IgA isotype, either spontaneously (B-1a) or in response to T-independent antigens (B-1a and B-1b). Marginal zone B cells produce mostly IgM in response to T-independent antigens.

B-1 cells represent a distinct population of B cells that arises during fetal development (33, 34). They are mainly found in the peritoneal and pleural cavities of mice and are rare in lymphoid organs and blood (Figure 1) (35). B-1 cells generally express germline-encoded, polyreactive IgM and IgA antibodies with limited V-gene segment usage, and are activated by T-independent antigens, such as LPS (TI-1) or multivalent antigens (TI-2) (36–38). In mice, B-1 cells can be further divided into B-1a and B-1b according to the expression of CD5 (CD5+ or CD5−, respectively). B-1a cells have been proposed as a major source of natural autoantibodies (37–40). These low-affinity polyreactive antibodies can be secreted spontaneously and are important in the clearance of apoptotic debris. They also contribute to protection against pathogens such as *Streptococcus pneumoniae* and influenza (41, 42). B-1b cells respond primarily to T-independent antigens (TI-1 and TI-2) and generate IgM memory cells, which contribute to protection against reinfection with *Borrelia hermsii, S. pneumoniae*, and Salmonella (19, 41, 43–45).

B-1 cells are poor at forming GCs (46); however, class-switched, somatically mutated B-1 antibodies showing evidence of antigen selection have been isolated from humans (47). Although elevated numbers of B-1 cells are present in some lupus-prone mouse strains (36, 48), there is not a clear association with SLE (49, 50).

### Marginal Zone (MZ) B Cells

Marginal zone and follicular (FO) B cells differentiate from transitional B cells and both can participate in T-dependent and T-independent immune responses. MZ B cells are located in the MZ of the spleen, where they can serve as a first line of defense to T-independent and blood-borne antigens, such as lipopolysaccharide from bacteria (51). They are characterized by high responsiveness to TLR activation, as well as a preactivated state with high expression of complement receptors and costimulatory molecules. Due to these characteristics, they are known for their ability to quickly differentiate into PCs in response to T-independent antigens (51–53). MZ B cells do not require cognate T cell help, as soluble factors such as cytokines and costimulation derived from DCs, neutrophils, iNKT cells, and T cells, can also lead to their activation, CSR, and differentiation into PCs (28, 31, 54, 55).

### FO B Cells

Follicular B cells are migratory cells that move between lymph nodes, splenic follicles and the circulation until they interact with antigen. While MZ B cells are specialized in the response to T-independent antigen, they can also transport these antigens to the follicles and transfer such antigens to FO B cells (56, 57). However, compared to MZ B cells which become blasts within 24 h of mitogen activation, FO B cells do not show blast formation in response to mitogen, due to their requirement for cognate T cell help, and therefore the contribution of FO B cells in T-independent responses is probably limited (51).

## T-Cell-Dependent Activation of B Cells

Although there are models where T-independent responses can contribute to lupus in mice (58, 59), the majority of studies in lupus-prone mice and SLE patients suggest that T-dependent responses are the main driver of the disease. Therefore, we will focus on T cell-dependent responses for the remainder of this review. Here, we will first discuss the initial activation of B cells in a T-dependent response, including the cell fate decisions into either the EF or the GC pathway, followed by a discussion of the PC differentiation pathways after they diverge.

**Figure 2:**
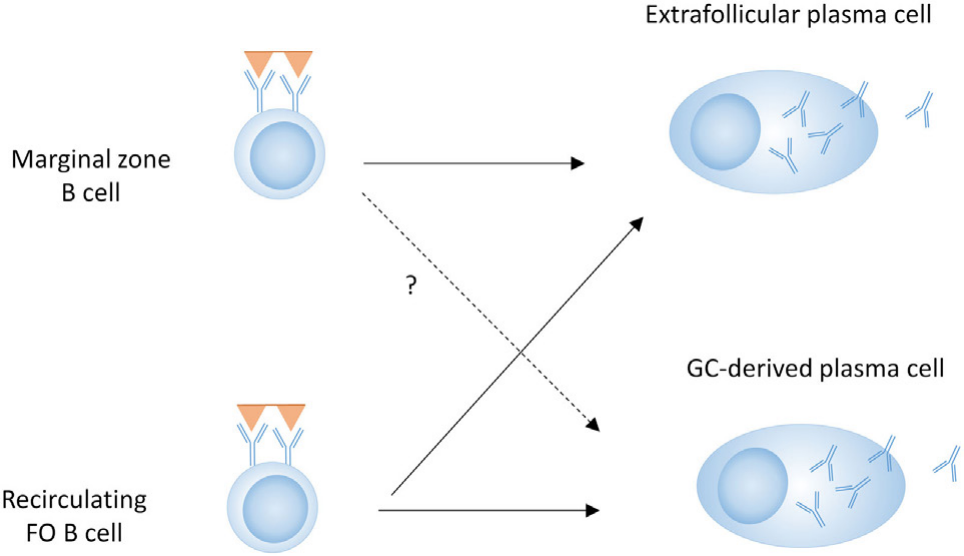
B cell subsets in T-dependent plasma cell (PC) development. Both marginal zone B cells and follicular (FO) B cells can contribute to the extrafollicular (EF) PC response, whereas the germinal center (GC) response and subsequent PC differentiation is predominantly driven by FO B cells.

T-dependent responses are thought to be dominated by FO B cells, although MZ B cells can migrate to the T–B border, activate T cells, and enter a GC (60–63). Although it is unclear how much they contribute to class-switched GC-derived antibody responses, MZ B cells can certainly contribute to T-dependent EF responses (Figure 2) (64, 65). MZ B cells can also capture and deliver blood borne antigens to the follicles, thereby enhancing T-dependent FO responses (66). This indicates that although MZ B cells do not require cognate T cell interactions for their differentiation into PCs, they can still participate in T-dependent responses (Figure 2).

Recirculating FO B cells are activated by antigen in peripheral tissues or in lymphoid tissue where they encounter soluble antigen or antigen arrayed on follicular dendritic cells (FDCs). Activated B cells upregulate chemotactic factors (CCR7, EBI2) that favor their migration to the B–T border in the lymph nodes or spleen (67, 68). At the same time, CD4+ T cells are activated by DCs in the T cell zone, and these activated T cells will also migrate to the B–T cell border. The differentiation of Th cells [Th1, Th2, Th17, T follicular helper cells (Tfh)] is determined by T cell–DC interactions and is driven by the engagement of pattern recognition receptors by pathogens- or damage-associated molecules (69). At the T–B border, cognate interactions between antigen-specific B and T cells drives initial proliferation, and some B cells will undergo immunoglobulin CSR under the influence of T cell-derived cytokines (60, 70–73).

After activation, B cells can diverge into EF PCs and GC B cells. While it is well known which transcription factors drive the differentiation into GC B cells versus EF PCs, whether there is direct competition between EF and GC differentiation has not been fully demonstrated. Since B cells can proliferate at the T–B border prior to making cell-fate decisions, it is possible that the distinction between cells with the same high affinity that enter an EF or a GC pathway is partly stochastic and that the same B cell clone can be found in both pathways (74, 75).

Under non-competitive conditions, low-, medium-, or high-affinity B cells can all seed a GC, whereas the low-affinity cells are unable to generate an EF antibody response (76), due to failure to expand low-affinity EF PCs rather than a lack of initiation of PC differentiation (75). In contrast, under competitive conditions, low-affinity cells compete with high affinity cells and are unable to expand or enter a GC response (77). The advantage of high affinity B cells in each response can be at least partly explained by the degree of T cell help that is received, as high affinity B cells stimulated with antigen express higher amounts of MHC class II in the membrane, and are able to present more peptide to T cells, establishing a more effective immunological synapse (77), which can affect both EF and GC responses.

Although TLR activation during T-independent responses clearly increases the magnitude of the EF response, TLR ligation in T-dependent responses can enhance both the GC response and the EF response (22), in a B cell-extrinsic or -intrinsic manner (78).

## PC Differentiation

### PC Differentiation in EF Responses

As EF PCs can provide an initial wave of secreted antibodies during the first week of an infection, these cells are an important part of the initial antibody response against pathogens (11). During expansion at the T–B border, those cells destined to become EF PCs will upregulate Blimp-1, CD138, and CXCR4 (79). Differentiation of EF PCs is driven by T–B interactions with a specific subset of Th cells that resembles Tfh cells but is located in EF areas. These Th cells are dependent on Bcl-6 and Stat3, and their interaction with B cells is mediated through CD40-CD40L and inducible T-cell costimulator (ICOS)–ICOSL interactions as well as cytokines, such as IL-21 (75, 79, 80). These interactions lead to heavy chain CSR as well as initiation of PC differentiation (Figure 4).

Under the influence of CXCR4, EF PCs migrate to the red pulp, where they can further proliferate and differentiate in EF foci. Proliferation is driven by BCR signaling, as cells with high affinity BCRs have increased proliferation and decreased apoptosis compared to cells with low affinity BCRs (75). Cofactors, such as CD19 and other molecules that enhance BCR signaling, can enhance EF proliferation. Lyn, an inhibitory molecule in the BCR signaling pathway, can diminish proliferation while in fact driving terminal PC differentiation (16). After the proliferative stage, PBs will differentiate further into PCs, characterized by higher expression of Blimp-1, further loss of MHC class II and costimulatory molecules. However, as discussed below, many EF PBs are short-lived and die prior to full differentiation into PCs (75). Some, however, complete their PC differentiation, after which they can survive in specialized niches in the spleen or bone marrow (BM) (12, 14).

Recent evidence suggests that several characteristics previously attributed to GC responses can also occur in EF responses. This includes the formation of memory B cells, CSR, SHM, and induction of long-lived PCs (discussed below). Whereas the previous understanding was that memory B cells are generated only in the GC, memory cells initially appear in blood before GC formation (81), and Bcl-6-deficient mice, which are unable to form GCs, generate memory cells (82). Most of these are non-mutated and IgM + memory cells, suggesting an EF origin at least for some memory cells. Although CSR and a low degree of SHM occur during early B–T interactions at the T–B border, continued AID expression and affinity maturation in EF sites has been observed (83).

### GC Responses

Germinal centers are areas of T-dependent B cell development in spleen, tonsils, lymph nodes, and Peyers’ patches. FDCs are important for normal splenic architecture and B cell development, as well as for maintaining the structure and function of the GC (84). Importantly, they capture antigen in immune complexes and retain the antigen in native form. Antigens are presented by FDCs on the cell surface (85). Several sequential events are involved in the formation of the GC. At the T–B border, B cell and T cell encounter with antigen stimulates formation of a GC (86), and migration from the T–B border into the follicle is mediated by CXCR5 (87). B cells receiving more T-help are more prone to differentiate into GC B cells (88, 89), and T cells differentiate into Tfh under the influence of B cell costimulatory molecules, including OX40L and CD80, which are essential for the maturation of Tfh cells (90, 91). The transcription factor Bcl-6 is required for the development of both GC B cells and Tfh cells (92). Tfh cells are specialized T helper cells that are involved in the selection and survival of B cells in the GC. The canonical costimulatory signal involved in the B–T cell interaction in the GC is CD40–CD40L (93, 94), but other signals such as ICOS–ICOSL, and IL-21 produced by Tfh cells are also required (95, 96).

The structure of the GC, with a light zone and a dark zone, aligns with the processes of SHM, affinity maturation, and selection (Figure 5). B cells in the light zone are referred to as centrocytes. They interact with FDCs through antigen and with Tfh cells through MHC–peptide interactions (97). Those B cells which make stronger interactions with Tfh cells, due to an increased T cell receptor peptide–MHC interaction, are positively selected and enter the dark zone where their proliferation is greater (98, 99). As more antigen is added, the population of B cells with BCRs that bind antigen sufficiently to induce positive selection increases (99). Positive selection in the light zone is important as it leads to GC B cells with the greatest affinity for antigen. Interactions between Tfh cells, antigen, and B cells in the light zone determine the extent of proliferation in the dark zone (99). Fewer cells move from the light zone to the dark zone than from the dark zone to the light zone indicating that selection occurs in the light zone (99, 100).

The dark zone is the location where the most active proliferation of GC B cells takes place, as all GC B cells that are in G2 or M phase are in the dark zone; however, S phase cells are present in both the light zone and dark zone (100). Proliferation can occur under the influence of mTORC1 kinase, which activates the metabolic program that permits proliferation of B cells in the dark zone (98). After positive selection in the light zone and while undergoing proliferation in the dark zone, SHM occurs to effect a process called affinity maturation. During this process, point mutations occur in the BCR which affect its affinity for antigen. When the B cell returns to the light zone, the B cells that have undergone mutations to enhance affinity for the antigen are preferentially selected (101). A stronger interaction with Tfh cells in the light zone allows the B cell to undergo more rounds of proliferation in the dark zone. Therefore, each time the cell divides and more mutations are acquired, more affinity maturation can occur for B cells that were most positively selected for in the light zone (99).

Negative selection also occurs in the GC. B cells with weak affinity for antigens in the GC, or autoreactive B cells recognizing ubiquitously expressed self-antigens are eliminated (102, 103). Proposed mechanisms for the negative selection of these B cells are Fas-mediated apoptosis of cells that fail to bind antigen, failure to receive continuing T cell help, or the activity of T follicular regulatory cells (Tfr) (102). A recent study, however, suggests that negative selection primarily occurs in cells with an unproductive BCR as a consequence of SHM rather than in cells with lower affinity (104).

### PC Differentiation in the GC

Both memory B cells and PCs arise from the GC, and many studies have examined the factors that determine if a given B cell will become a memory B cell or a PC. High affinity GC B cells become PCs, while lower affinity GC B cells become memory B cells (105–107). The initiation of PC differentiation in the light zone requires strong affinity for antigen; further differentiation in the dark zone requires help from Tfh cells (108). Light zone B cells become memory B cells early in the GC reaction, while PCs are formed later (105, 109). Preventing apoptosis in the GC allows for lower affinity B cells to become memory B cells but does not change the development of PCs, further suggesting that selection of B cells into the PC population is dependent on high affinity for antigen (106).

Certain cytokines favor the development of PCs. Among them, IL-21 is the most potent inducer of PC differentiation from memory and naive B cells (110, 111). This cytokine is produced by Tfh cells in the GC and activates the JAK1/3 STAT3 pathway. IL-21-deficient mice are unable to generate fully functional GCs. Without IL-21 or Tfh cells, PC formation is disrupted, affinity maturation does not occur, and the population of memory B cells is expanded (91, 96, 110).

Toll-like receptor ligands also enhance GC responses through both DCs and B cells (21, 78, 112). Whereas soluble TLR ligands can enhance GC responses through an effect on DCs, an antigen that can trigger both endosomal TLRs and BCRs can enhance the IgG antibody response in a B cell-intrinsic manner (21). This probably reflects the requirement for BCR-mediated uptake of ligands for endosomal TLRs in this process, and explains why some studies reported no effect of TLR signaling on GC responses induced with LPS (113, 114). B cell-intrinsic MyD88 signaling specifically enhances the formation of GC B cells, affinity maturation, and CSR in response to the TLR-9 ligand CpG coupled to the hapten NP, without affecting the number of PCs. In contrast, MyD88 signaling in DCs contributes to PC differentiation without affecting affinity maturation (78).

Different transcription factors are involved in the differentiation of PCs and memory B cells. Bach2 is reported to be important for selection of GC B cells into memory B cells; in the light zone, B cells with lower affinity for antigen have higher expression of Bach2, probably due to a lower degree of T cell help in those cells (105, 115). In addition to Bach2, ABF-1 leads to memory B cell differentiation and prevents PC differentiation (116). The transcription factors Blimp-1, XBP-1, and IRF4 are all involved in PC differentiation (117–119). Blimp-1 leads to decreased expression of genes involved in B cell signaling pathways including Pax5, which in turn leads to increased expression of Blimp-1 and XBP-1. This feed-forward mechanism is needed for PC differentiation (120, 121). Whereas Blimp-1 is required for PC differentiation, XBP-1 is more specifically needed for the unfolded protein response that is required for the production of high amounts of immunoglobulin in PCs (122).

It has been recently reported that PC differentiation is initiated in light zone B cells after which they migrate to the dark zone to further differentiate (108). Together with simulation data, this suggests that PCs exit the GC through the dark zone (123). Similar to the EF response, GC-derived PCs are characterized by a proliferative PB stage. Proliferating PBs have been reported in the dark zone of the GC, as well as the T–B border directly adjacent to the GC (123, 124), and their proliferation decreases as they migrate further from the GC, and is completely lost as they reach the medulla of the lymph node or the splenic red pulp (124). This suggests that proliferation of GC-derived PBs occurs during their transit out of the GC, at distinct locations from EF PBs, which proliferate in EF foci mainly in the red pulp of the spleen or the medulla of the LN. Some GC-derived PCs migrate to the red pulp in the spleen or the medullary cords in the lymph nodes, and others migrate through the blood to the BM (14, 81). Their exit out of the secondary lymphoid organs occurs prior to completion of their differentiation, as circulating PBs that arise in GC responses in humans show signs of recent proliferation such as expression of Ki67 (14, 125).

### PC Survival

Two studies in the late 1990s showed the existence of long-lived PCs, disputing previous thinking that PCs were short-lived (126, 127). A more recent study showed that 10 years after vaccination, long-lived PCs were still present in the BM, despite memory B cell depletion (128). Another study shows the survival of these long lived PCs despite CD19 directed CAR T cell therapy (129). These PCs have become a challenge in treatment of SLE, as they are often not eliminated by traditional therapies (130, 131). Although most evidence suggests that selection of PCs into the long-lived PC pool is dependent on extrinsic factors (132, 133), there is some evidence that B cell-intrinsic factors are also involved (134, 135). Identification of intrinsic factors leading to long-lived PC survival could represent therapeutic targets for SLE and other autoimmune diseases.

Plasma cell survival depends on cytokines secreted by stromal cells and eosinophils in the BM (136, 137), but they can also survive in the spleen or other organs, particularly under inflammatory conditions. PCs can survive anywhere as long as sufficient survival factors are present (138), but niches have the capacity to support only a limited number of PCs (132). Two related factors important for survival of PCs are BAFF and APRIL, which act through binding to TACI and BCMA (139–141). Both cytokines are anti-apoptotic and increase PC survival (140). A study in autoimmune thrombocytopenia suggests that an increase in BAFF caused by B cell depletion promotes differentiation of short-lived PCs into long lived PCs in the spleen (142, 143). Other molecules which can enhance survival of PCs are IL-6, VCAM-1, CXCR4, and CD28 (11, 136, 144).

CD93, a C1q receptor on B cells, is needed for the survival of PCs in the BM and is expressed only by a subset of PCs in mice (145). Induction of CD93 expression may, therefore, be an example of a B cell-intrinsic factor that contributes to PC survival.

Despite the traditional paradigm mentioned above, there are descriptions of long-lived PCs in T-independent responses, T cell-deficient, and GC-deficient mice, with survival up to at least 100 days (12, 132, 146, 147). In addition, PCs exit the GC as PBs, and require a survival niche for full differentiation. As many of them fail to find the appropriate niche, not all GC-derived PCs are long-lived (12). As far as we know now, transcription factors that drive PC differentiation in each response are similar, and it is not clear if all PCs have the potential to become long-lived or whether some are selected, preferentially in the GC, to become long-lived, and whether this is accompanied by altered expression of key survival molecules and transcription factors that drive this distinction.

## Tolerance

### Tolerance in EF Responses

As autoimmunity has been traditionally thought to arise through the GC, tolerance checkpoints in EF responses have not been extensively studied. Whereas the fast rate of the EF response is needed for adequate responses against pathogens, it also limits the time window for tolerance checkpoints. Therefore, it is likely that autoreactive B cells can be activated during EF responses, either through direct activation by self-antigen in an inflammatory milieu, cross-reactivity with foreign antigen, or through TLR ligands or cytokines (148). However, even if autoreactive PCs are generated in EF responses, they are mostly short-lived limiting the inflammation and tissue damage that is induced by autoantibodies. Therefore, the transient nature of the EF response may itself be a tolerance mechanism.

In addition to the short-lived nature of the response, tolerance in EF responses can be maintained through the balance between IgM and other (more pathogenic) isotypes. As most EF PBs secrete IgM, even though some CSR can occur, the balance between IgG and IgM that is generated in EF responses may result in prevention of autoimmunity, through downregulating myeloid cell activation in a LAIR-1 dependent fashion and minimizing local inflammation (3, 5, 149). In addition, sialylation of IgG antibodies, which occurs in T-independent responses can also contribute to tolerance, as these antibodies have lower pathogenicity, at least in the context of rheumatoid arthritis [(150), p. 296; (151), p. 429].

T cell help during initial activation might play a role in tolerance in EF foci, where class-switched EF responses can occur through cytokines secreted by bystander T cells or non-T cells. Since there is no requirement for cognate T cell help, the T cell repertoire is unlikely to restrict autoreactivity in the EF response.

### Tolerance in GC Responses

Although the mechanisms of central tolerance preclude many autoreactive B cells from entering GCs, self-reactive B cells developing in the BM can bypass tolerance mechanisms. This may occur if they are reactive to monovalent antigen, if their affinity for antigen is below a certain threshold, or if they are present in an inflammatory milieu (152, 153). Therefore, it is normal to have circulating autoreactive mature (naive) B cells (154).

Importantly, however, mechanisms of peripheral tolerance are also in place to further eliminate autoreactivity. Mature self-reactive B cells can be thwarted from entering the follicle and be induced to become anergic (155). Still, some self-reactive B cells are able to enter the follicle. Evidence also suggests that autoreactive B cells that were initially excluded from the follicle can later be recruited into the GC, at which point these cells undergo SHM which may remove autoreactivity (156, 157), but can also lead to enhanced self-reactivity (153, 158). One important tolerance mechanism is the short lifespan of these B cells without mitogenic stimulation. Thus, only in an inflammatory milieu are these cells likely to access a GC response.

B cells that acquire autoreactivity in the GC must be eliminated or prevented from becoming PCs. In the GC itself, several tolerance mechanisms have been described, including apoptosis and receptor editing. However, a recent study showed that autoreactive GC B cells are not strongly selected to undergo apoptosis, perhaps because so many autoreactive B cells are cross-reactive with an eliciting antigen (104, 159–163). It is conceivable that these tolerance mechanisms are initiated by lack of cognate T cell help. As described, T-cell help is needed for positive selection of B cells into PCs in the GC, and without this help, self-reactive PCs will not develop (100, 102, 152, 153). Thus, although self-reactive memory B cells can develop, the requirement for Tfh cells and FDCs that recognize or present the autoantigen makes it more difficult for non-cross-reactive autoreactive PCs to develop. T cells recognizing foreign antigen may be able to stimulate autoreactive GC B cells, if the BCR crossreacts with the eliciting antigen or an antigen present in a multimolecular complex with the eliciting antigen.

**Figure 3:**
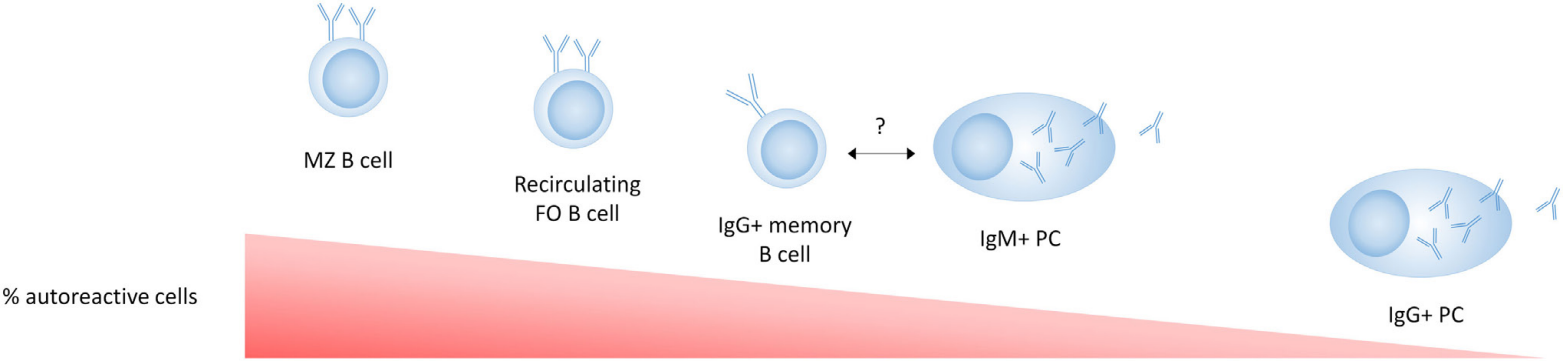
Tolerance in mature cell subsets in healthy individuals. B-1 cells are not shown in this figure as their relative autoreactivity compared to the other subsets is not exactly known. Marginal zone (MZ) B cells are enriched for autoreactive B cell receptors (BCRs) (polyreactive or antinuclear) compared to follicular B cells. IgG + memory B cells have a lower frequency of autoreactivity compared to naive B cells, suggesting a tolerance checkpoint in the germinal center. The autoreactivity in IgM plasma cells (PCs) has not been directly reported, but the fact that IgM autoantibodies are commonly found in healthy individuals and have an anti-inflammatory role, suggests that some autoreactive IgM + PCs are present and more common than autoreactive IgG + PCs. The low frequency of autoreactive BCRs in IgG + PCs compared to IgG + memory B cells suggests an additional strong tolerance checkpoint that prevents the development of serum IgG autoantibodies.

As IgG + memory B cells in healthy individuals have a much higher frequency of self-reactivity than IgG + PCs (164), an additional tolerance checkpoint must exist that prevents the differentiation of autoreactive PCs in addition to a tolerance checkpoint in GC B cells (Figure 3). Interestingly, switched PCs maintain expression of MHC class II and the antigen presentation machinery required for cognate T cell interactions at least until they are no longer proliferating, suggesting that this stage of PC differentiation may represent a T cell-dependent tolerance checkpoint (165). Although this has not been extensively studied, Th cells are required for the completion of GC-derived PC differentiation (103), and PCs can undergo cognate T cell interactions after their migration out of the GC at the T–B border (165). This suggests that a lack of T cell help may prevent the terminal differentiation of autoreactive PCs or that autoreactive B cells committed to becoming PCs are more susceptible to apoptosis or receptor editing than B cells committed to a memory pathway.

## PC Differentiation in SLE

### EF PC Differentiation in SLE

**Figure 4:**
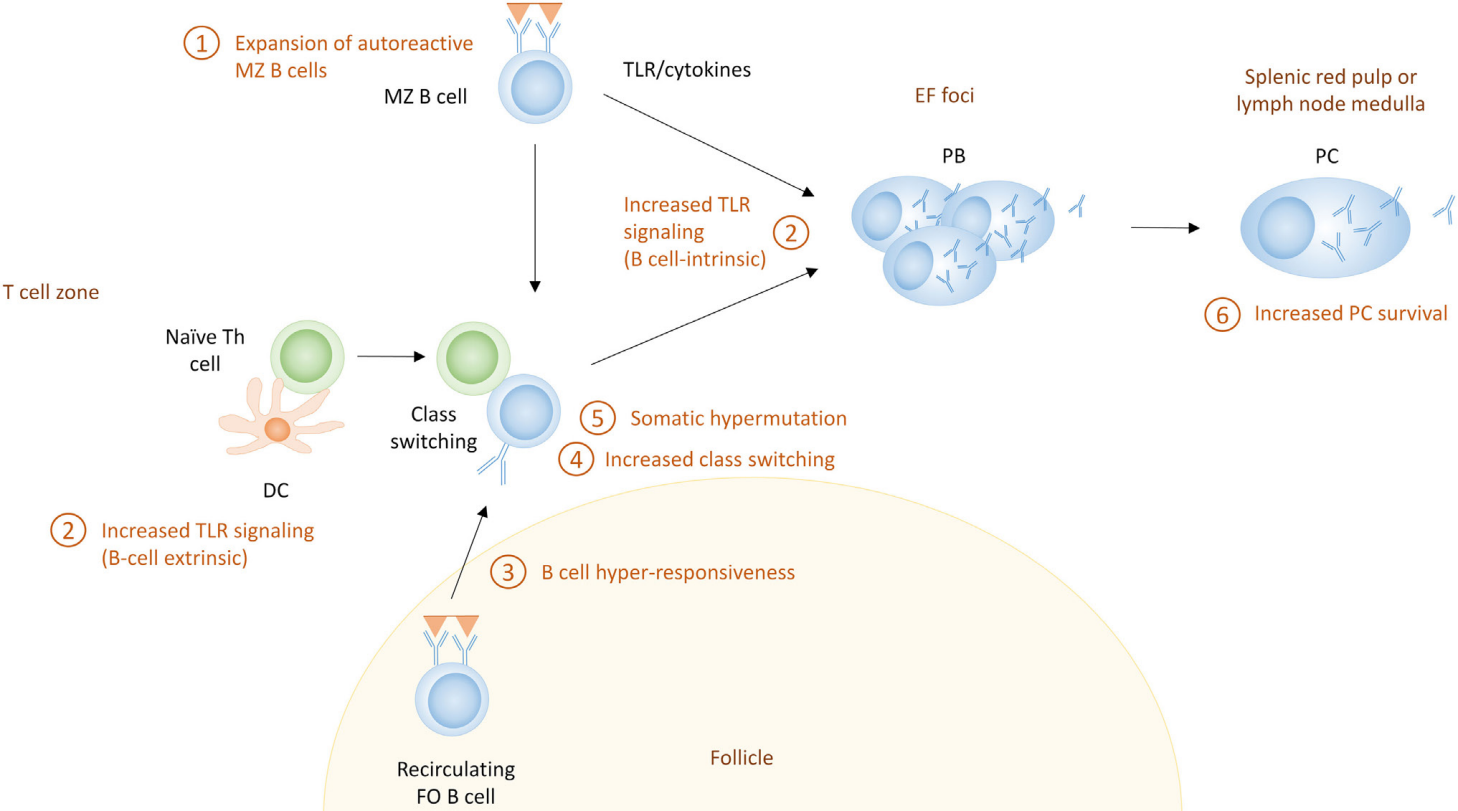
The extrafollicular (EF) pathway for the generation of autoreactive plasma cells (PCs) in systemic lupus erythematosus. Shown here are the potential mechanisms that can contribute to enhanced extrafollicular PC responses. These include (1) expansion of marginal zone (MZ) B cells, which often exhibit autoreactive receptors; (2) increased Toll-like receptor (TLR) signaling, which can directly activate B cells (B cell-intrinsic) or can enhance Th responses through their effect on dendritic cells (B cell-extrinsic); (3) B cell hyperresponsiveness, which can affect the activation of follicular B cells; (4) increased class switch recombination which can lead to more pathogenic IgG autoantibodies; (5) somatic hypermutation which can lead to affinity maturation of autoreactive PCs; and (6) increased PC survival.

Because there are no definite markers that discriminate PCs based on their pathway of differentiation, it is hard to establish the pathway through which they were derived, especially in humans where access to lymphoid organs is limited. In addition, most studies discriminating EF responses from GC responses use acute immunization models, and it is not clear if all the paradigms that have been proposed for the distinction between EF and GC responses apply in the chronic immune activation present in autoimmune conditions. Although EF PC differentiation in autoimmunity has not been emphasized, recent studies indicate this pathway may have a specific role in autoimmunity (125, 166, 167). MRL/lpr mice exhibit EF PC generation, although they have increased formation of spontaneous GCs as well (166, 168, 169). In humans, recent research supports that a large proportion of the PCs in some SLE patients are clonally related to naive cells, suggesting an EF origin (125). Here, we propose mechanisms which can lead to enhanced EF responses in SLE (Figure 4).

#### Expansion of MZ B Cells

Marginal zone B cells are expanded in several lupus-prone mouse strains. In humans, the characterization of MZ B cells is much more complicated [reviewed in Ref. (13)], and it is unclear if SLE patients also have an expansion of this population. However, the high BAFF levels often present in SLE patients would support MZ expansion. Mice overexpressing BAFF develop an SLE-like phenotype that is characterized by a high titer of class-switched autoantibodies and PCs, in a T cell-independent manner (59). A preference for autoreactive B cells to differentiate into MZ B cells compared to FO B cells has been described in mice (170–173), and MZ B cells can differentiate directly into IgG + PCs in EF responses (174, 175). Therefore, development of serum autoantibodies in some lupus-prone mice has been attributed to MZ expansion and activation, although some studies have challenged this paradigm (176–180).

#### Enhanced TLR Signaling

Another mechanism by which EF PC responses in SLE may be altered is through enhanced TLR signaling. MyD88-deficient MRL/lpr mice develop lower autoantibody titers and are protected from disease (181, 182), suggesting a role for TLRs in EF responses in MRL/lpr mice. Although TLR-7 or -9 deficiency each diminished the production of specific types of autoantibodies in MRL/lpr mice, only TLR-7 deficiency diminished lymphocyte activation, IgG production, and kidney disease (183, 184). This suggests that although each receptor can enhance EF responses, only TLR-7 induces the production of pathogenic antibodies, or permits the inflammatory response needed to cause disease.

Besides a B cell-intrinsic role of TLR signaling, B cell extrinsic TLR signaling can also enhance T–B interactions through the increased activation of DCs (185). Enhanced T–B interactions in this situation have the potential to enhance T-dependent PC differentiation in both EF and GC pathways. B cells as well as myeloid cells from SLE patients have increased expression of TLRs, and SLE patients may have increased proinflammatory responses to TLR ligands (186), which can contribute to stronger T-independent and T-dependent EF responses.

#### B Cell Hyperresponsiveness

A well-known feature of SLE is B cell hyperresponsiveness, which causes increased signaling upon BCR ligation by antigen (187, 188). The increased signaling can derive from increased activity of signaling molecules in the BCR pathway (many of which are genetic risk factors for SLE; discussed below) (189), or through a synergy between BCR triggering and other signaling pathways, such as TLR, BAFF, and type I IFN, which can each lower the threshold for B cell activation through the BCR and contribute to the activation of B cells (59, 190, 191). Type I IFN is necessary for a complete response after BCR/TLR7 stimulation, and increments in type I IFN can overcome tolerance that normally occurs after repetitive stimulation of TLRs (192). The fact that high affinity B cells are more prone to expansion at the EF PB stage (75, 76) suggests that the increased BCR signaling that occurs in SLE may preferentially stimulate EF responses.

#### Increased CSR

Increased CSR in EF responses is another feature of SLE that may contribute to enhanced pathogenicity of EF PCs, in particular if the balance between protective IgM and pathogenic IgG is altered. Increased CSR has been described both in lupus-prone mouse models as well as SLE patients (193). In particular, a special subset of EF T cells in the MRL/lpr mice has been described to contribute to the expansion of class switched IgG + EF PCs. These EF T cells are dependent on Bcl-6, Stat3, and ICOS, and they mediate IgG CSR through CD40–CD40L interactions and IL-21 (167, 168). A similar subset of T cells has been described in EF responses in non-autoimmune mice, although there they localized at the T–B border, and it is not clear if they migrate to EF foci as well (80). EF Th cells express CXCR4, as opposed to Tfh cells which express CXCR5 (or both) (194, 195). While the EF Th cell subset is present in MRL/lpr mice which have a dominant EF phenotype, mice with a more pronounced GC pathway, such as NZB/W, have a more mixed T cell phenotype (168). IgG CSR in MRL/lpr mice, as well as in graft versus host-mediated autoimmunity, is almost completely dependent on ICOS, as ICOS-deficiency leads to lower expression of CXCR4, as well as diminished secretion of IL-21 (167, 168). T-independent factors can also increase EF CSR in SLE. In AM14 rheumatoid factor transgenic MRL/lpr mice, T cells are required for the spontaneous production of rheumatoid factor, but not when B cells are exposed to chromatin immune complexes which will trigger both the BCR and TLR (196).

Increased CSR has been described in circulating PBs of SLE patients, and at least some of these have low mutation rates, suggestive of an EF origin (125). However, all EF-derived PBs need not have low mutation rates. Factors that increase CSR in EF PCs in mice, such as IL-21, are increased in SLE patients (197), and factors that mediate T-independent CSR, such as TLR signaling and the myeloid-derived cytokine BAFF, are also increased in SLE (198). It is therefore conceivable that SLE patients can exhibit increased CSR in EF responses.

#### Increased SHM

Besides the increased CSR in EF PCs in MRL/lpr mice, SHM has also been shown to occur in EF foci, probably under the influence of EF Th cells. However, SHM can also occur in response to chromatin immune complexes in a T-independent manner (166, 168, 196, 199). This SHM potentially leads to affinity maturation (although probably to a lesser extent than in the GC) in autoreactive EF PCs, but no mechanism has been described for antigen selection and affinity maturation in EF responses.

### GC Responses in SLE

**Figure 5:**
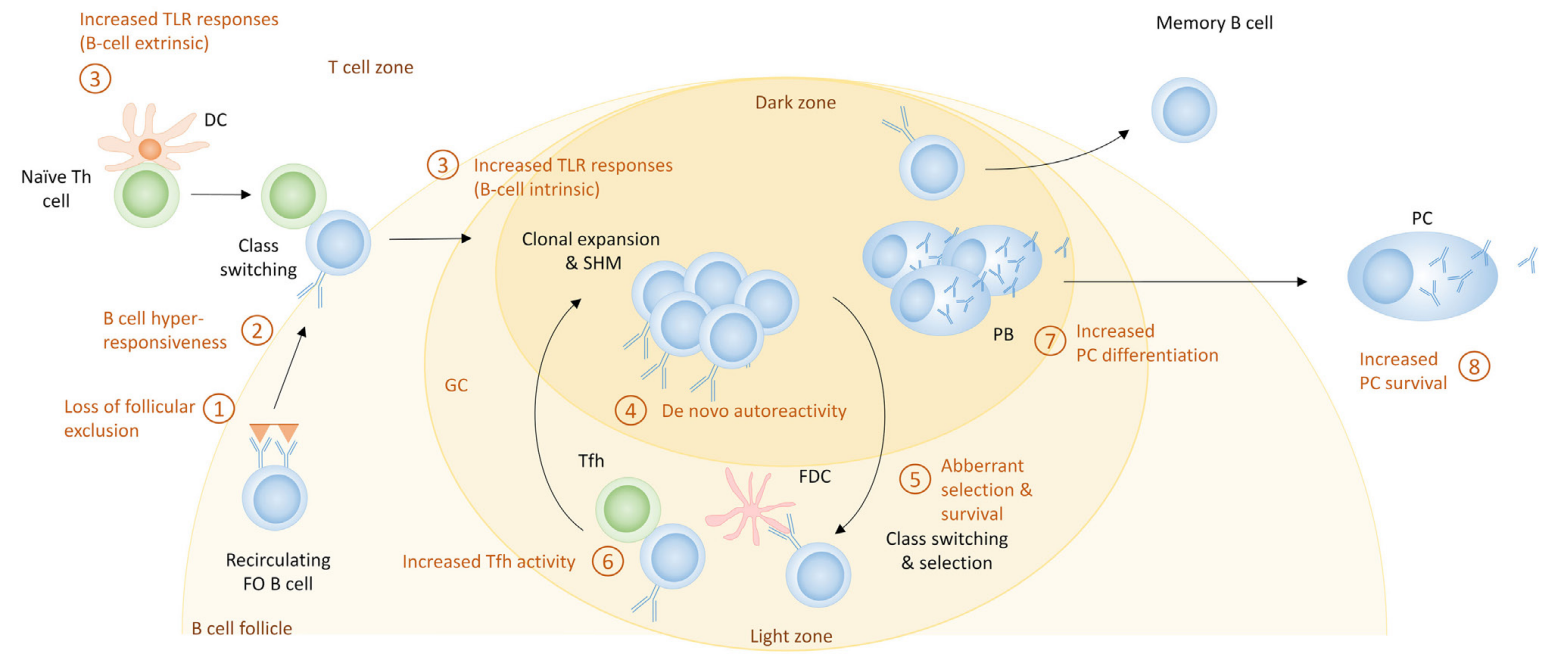
The germinal center (GC) pathway for the generation of autoreactive plasma cells (PCs) in systemic lupus erythematosus. Shown here are the potential mechanisms that can contribute to enhanced PC differentiation during GC responses. These include (1) loss of follicular exclusion, which can lead to recruitment of autoreactive B cells into GC responses; (2) B cell hyperresponsiveness which can affect the activation of follicular B cells; (3) increased Toll-like receptor (TLR) signaling, which can affect initial activation of B cells as well as the GC response itself; (4) *de novo* autoreactivity, generated through somatic hypermutation (SHM) and leading to the generation of autoreactive GC B cells from non-autoreactive precursors; (5) aberrant selection and survival, which can diminish tolerance mechanisms; (6) increased T follicular helper (Tfh) activity, which can increase the extent of GC responses as well as PC differentiation; (7) cell fate decisions that increase PC differentiation; and (8) increased PC survival.

Germinal center responses are well known to be increased in lupus-prone mice, and SLE patients have increased numbers of circulating pre-GC B cells, switched memory B cells and Tfh cells, suggestive of enhanced GC responses (169, 200, 201). Given that IgG anti-DNA autoantibodies which are considered to be pathogenic in SLE show evidence of SHM (202), the production of autoreactive PCs by SHM of nonautoreactive naive B cells within the GC has been considered an important contributor to the development of SLE in both mice (203) and humans (204). The following mechanisms can contribute to GC-derived autoreactive PCs (Figure 5).

#### Loss of FO Exclusion

In normal conditions, autoreactive naive B cells undergo anergy that leads to FO exclusion and prevents their recruitment into GC responses. However, in SLE, these B cells are able to enter the GC, and continue their differentiation into PCs (155, 205).

#### B Cell Hyperresponsiveness

As discussed above, B cell hyperresponsiveness can enhance EF responses and may also potentiate GC responses (206). Hyperresponsiveness could lead to increased positive selection in the light zone and subsequent proliferation in the dark zone, thereby amplifying GC responses (207).

#### Increased TLR Responses

Toll-like receptors, which are involved in the EF pathway, also have a role in the GC response. Loss of MyD88 causes a loss of GC formation; interestingly, this alteration may be attributed to the function of TLR-7 and not TLR-9 (208, 209). Increased function of TLR-7 causes an increment in spontaneous GC formation and an autoimmunity phenotype (208). B cell-intrinsic MyD88 signaling specifically enhances GC responses when antigen and TLR ligand are coupled; self-antigen that can trigger both TLRs and the BCR will presumably have the same ability. It was recently demonstrated that FDCs, which are crucial for the maintenance of GCs and FO architecture, express type I IFN through a TLR-7 pathway upon internalizing complement-opsonized self-immune complexes through the complement receptor CD21 in the 564Igi RNP-specific lupus mouse model; this pathway is important for spontaneous GC formation and production of isotype-switched autoantibodies (210). 564Igi BM chimeras in which the recipient FDCs were TLR-7-deficient exhibited less autoimmunity. As many SLE antigens can activate TLRs (1, 190, 191), these represent potent pathways to amplify GC responses.

#### *De Novo* Autoreactivity

Although loss of FO exclusion can lead to recruitment of autoreactive naive cells into GC reactions, SHM of nonautoreactive B cells can lead to *de novo* autoreactivity in GC B cells (202, 211). Whether these cells are able to differentiate into PCs has not been reported, but most pathogenic antibodies in SLE show signs of SHM, and *de novo* autoreactivity explains a large fraction of the autoreactive IgG + memory cells in SLE patients (204, 212). These studies suggest that GC B cells retain autoreactivity generated through SHM.

#### Aberrant Selection and Survival of GC B Cells

Another mechanism for the generation of autoreactive PCs in the GC is increased survival of GC B cells. It has been shown that SLE patients have increased levels of BAFF (213). While BAFF has a large role in the EF pathway of differentiation of PCs, it also expands the Tfh cell population and promotes formation of GC and survival of B cells. This could be a contributing factor in allowing the breach of B cell tolerance seen in SLE patients (214, 215). As a result of increased BAFF, naive B cells with moderate affinity that would normally undergo apoptosis, may be rescued and enter a GC response.

#### Increased Tfh Activity

Another mechanism that leads to the development of autoreactive PCs in the GC is increased Tfh activity (216). One cytokine important for the development of autoreactive PCs is IL-21, a cytokine produced by Tfh cells (72). The number of Tfh cells as well as the level of IL-21 has been shown to be increased in lupus-prone mice and SLE patients (217, 218). IL-21 increases IgG PC number (72, 217), and Tfh cells can alter selection and allow the differentiation of autoreactive B cells into autoreactive antibody secreting PCs (219). In lupus-prone mice, OX40L expression by B cells contributes to the autoimmune phenotype, presumably through its effect on Tfh cells (90, 220).

#### Increased PC Differentiation

B cell hyperresponsiveness may also increase the generation of PCs in SLE by directing more B cells to undergo PC differentiation. SLE patients often have increased numbers of circulating PBs; in one study this inversely correlates with the number of CD27 + memory cells, suggesting a preferred differentiation pathway (221). Lupus-prone mice, including the ones that have a GC phenotype, have vast increases in their PC numbers, which exceeds the expansion of the memory compartment. This suggests that there may be preferential output of PCs from the GC in SLE.

### Increased Survival of PCs

Another possible mechanism for increased autoantibody titers is increased survival of PCs (Figures 2 and 3), which might occur if excess survival factors are present. Increased expression of the cytokines BAFF, APRIL, and IL-6 is present in lupus-prone mice (222, 223), suggesting that these cytokines can support enhanced PC survival. Although in healthy mice a limited number of PCs can survive in BM and spleen, these organs in lupus-prone mice gain additional capacity and exhibit an increased number of PCs (224). Both lupus-prone mice and SLE patients often exhibit hypergammaglobulinemia (225, 226), which may also be caused by an increased capacity to support PC survival in SLE patients. As both EF and GC-derived PCs can either stay in the spleen or stay in the lymph nodes or migrate to the BM, these factors will probably affect both types of PCs. In addition, several lupus-prone mouse strains have increased levels of CXCL12 in their inflamed kidneys, which may allow recruitment of PCs to this organ (226–228).

## Genetic Risk Alleles

Whereas both EF and GC pathways can lead to autoantibody production, we propose that genetic factors may cause a dominance of either of these pathways in individual patients. Approximately one hundred risk loci have been associated with SLE, and genes within these loci have been broadly cast into categories involving DNA degradation and clearance of apoptotic/cellular debris, innate immunity, including TLR and IFN signaling, and adaptive immunity (229). Some overlap or fall outside these categories, and others are undefined. Most variants are in non-coding regions that may alter expression levels that can determine the magnitude of a response in a cell-lineage and stage-specific manner. Several risk genes have been described to alter B cell selection, activation, differentiation and/or survival in a B cell-intrinsic fashion, including LYN, BLK, BANK1, PTPN22, TNFAIP3, TNIP1, CSK, and FCGR2B (189, 230). Although it is known that these risk alleles alter B cell signaling, their effect on PC differentiation has not been extensively investigated, and most of our understanding of the role of these genes in EF and GC responses derives from mouse models. Other risk alleles are involved in B–T cell interactions, memory or PC differentiation, and IFN/TLR signaling. For many of these, the functional consequence of the risk allele has not been determined. However, it is reasonable to ask whether these risk alleles may alter EF or GC responses.

Table 1 shows risk alleles that can alter B cell responses and subsequently PC differentiation. These risk alleles can function in a B cell-intrinsic or -extrinsic manner. We propose that some risk alleles, such as TLR7, FAS, IRF5, TNFAIP3, and TNIP1, can modify both EF and GC responses.

**Table 1:** The role of genes with risk alleles in EF and GC responses.

Gene	Function of gene	Function of risk allele	Potential role in EF	Potential role in GC	Reference
*HLA class II* genes	Antigen presentation	Presentation of self-specific T cell epitopes	May increase T-dependent EF responses	Expansion of Tfh, GC responses and PC differentiation	(103, 231, 232)
*TNFS4* (OX40L)	Costimulatory molecule on many cell types primarily interacting with OX40 on activated T cells promoting T cell functions, cytokine and Ab production, and PC generation	Most likely a response eQTL, as DNA heterozygous for the 5′ rs2205860 SNP had enhanced binding to NF-κB; no significant differences in basal expression in EBV-transformed cells or primary cells	Required for T cell-dependent EF Ab response driven by MZ DCs	Supports Tfh maturation in mice (B cell-intrinsic) and in humans (expression on myeloid APCs)	(64, 90, 220, 233, 234)
*CD80*	T cell costimulation through CD28, CTLA-4, PD-L1	Unknown	Little effect on Ab production by short-lived plasmablasts (in CD80−/− mice)	Increased maturation of Tfh and generation of long-lived PC	(91)
*LYN*	Src-family kinase that phosphorylates both activating and inhibitory receptors in B cells and myeloid cells. Its role in activating ITAMs is probably redundant with other Src family kinases, therefore its role in inhibitory receptors seems most crucial	Unknown SLE patients have decreased expression of Lyn in B cells	Lyn−/− mice have EF PC differentiation without GCs in some studies	Lyn−/− mice have spontaneous GCs in some studies	(187, 235, 236)
*BLK*	Src-family kinase that phosphorylates both activating and inhibitory receptors in B cells	Decreased expression in B cells, increased B cell activation	Increased TI IgG antibody responses in Blk+/− mice, with no effect on IgM	Increased numbers of switched memory cells in risk carriers suggests more active GC responses, but TD antibody responses in Blk+/− or −/− mice not affected	(237–240)
*BANK1*	Signaling molecule involved in BCR- and CD40-mediated signaling in B cells, positively regulates Ca^2+^ release in B cells, negatively regulates CD40-mediated signaling	Differential expression of two splice variants, but functional consequences unknown	Normal antibody responses to TI antigen, but the IgM response in TD responses was increased, possibly due to increased survival of EF PCs	Normal IgG antibody responses to TD antigen suggesting that there is no major influence on switched TD responses, although there is a slight increase in spontaneous IgG2a in Bank1−/− mice	(241–243)
*PTPN22* (Lyp)	Protein tyrosine phosphatase that has inhibitory function in B and T cell signaling	Risk allele has increased inhibitory function, causing decreased B cell activation, proliferation and signaling leading to impaired central B cell tolerance as well as impaired T cell responses	Impaired central tolerance; unclear if PTPN22 affects EF responses; deficiency of PEP (mouse ortholog) in mice did not alter spontaneous IgM and IgG3 levels, suggesting no effect on extrafollicular antibody production	Lower frequency of memory cells in risk allele carriers suggests that it may inhibit GC responses consistent with increased GCs and serum IgG in PEP−/− mice	(206, 244–247)
*TNFAIP3* (A20)	Negative regulator of NF-κB signaling in response to TLR, TNF, and CD40 signaling in B cells and other immune cells	Reduced expression in EBV transformed cells with one risk variant and reduced anti-inflammatory activity in transfected HEK cells with another risk variant	B cell-specific A20 deficiency in mice leads to alterations in the MZ compartment and consistently enhanced IgM production (spontaneous, as well as TD and TI immunizations), but no difference in IgG3	B cell specific A20 deficiency in mice leads to elevated numbers of GC B cells, and spontaneous IgG2 levels in old mice which deposited in kidneys; however, inconsistent effects on TD IgG production upon immunization in different studies	(248–253)
*TNIP1*	Ubiquitin-binding protein with diverse targets; interaction with TNFAIP3 negatively regulates NF-κB; also known to repress PPARs, which may increase B cell activity	Reduced expression in EBV-transformed B cells from H1 and H2 risk haplotypes; H1 contains coding SNP near a nuclear export sequence	Mutation of polyubiquitin binding site increased formation of EF PCs	Mutation of polyubiquitin-binding site induced spontaneous GC, increased TFH, CSR, and production of autoreactive Abs thru TLR-mediated NF-κB pathway	(254, 255)
*CSK*	Tyrosine kinase protein that phosphorylates Src family kinases leading to their inactivation. Src family kinases can act on both activating and inhibitory receptors in B and T cells	Increased expression in B cells, increased B cell activation (Lyn phosphorylation, Ca^2+^ mobilization), expansion of transitional B cells	Unknown, but increased signaling may enhance PC differentiation	Csk is low in memory cells but its function in GC responses is not known	(256)
*FCGR2B*	Inhibitory receptor for IgG on B cells and other immune cells	Impairment of receptor mobility, lipid rafts and inhibitory signaling	Enhanced antibody production upon TI immunization, although not observed in all studies	Enhanced GC responses in FCGR2B−/− mice. Spontaneous GC B cells have increased self-reactivity, but the checkpoint to PCs is still intact in FCGR2B−/− mice, so uncertain if autoreactive PCs in these mice are GC-derived	(150, 257–262)
*IRF5*	Production of type I IFN in response to TLR ligands, macrophage polarization, enhanced PC differentiation	Increased expression and activation in monocytes from SLE patients with the risk allele	IRF5−/− mice have decreased IgG1 responses upon TI immunization and have decreased PC numbers in MRL/lpr mice, suggesting that increased expression of IRF5 may enhance EF responses	IRF5−/− mice have diminished GC-derived antibodies, suggesting the IRF5 risk allele may enhance GC PC differentiation	(263–267)
*STAT4*	Transcription factor critical for myeloid and lymphocyte functions; major responder to IL-12; role in IFN-α signaling	Increased expression in PBMCs correlated with SNPs rs3821236, rs3024866 (both in the same haplotype block) and rs7574865 but not with other SNPs	STAT4−/− had no effect on antibody titers or pathology in EF model (MRL/lpr)	Regulates Tfh through Bcl-6 and T-bet in T cells; indirectly upregulates T-bet in B cells, which facilitates spontaneous GC; STAT4−/− reduced autoantibody production and glomerulonephritis in B6.TC model (Sle1,2,3 congenic)	(268–270)
*BACH2*	Transcriptional repressor that promotes CSR/SHM and is required for memory B cell differentiation	No expression differences associated with rs597325 in primary blood cell types	Deficiency increases IgM PC differentiation *in vitro*	Bach2 can enhance memory B cell differentiation while blocking plasma cell differentiation	(105, 271–273)
*PRDM1* (Blimp-1)	Transcription factor required for PC differentiation/transcription factor that alters DC function	Decreased expression of Blimp-1 in DCs leading to increased cytokine production (unknown function of risk allele in PCs)	Unknown	Expansion of Tfh and increased GC responses in DC-Blimp-1-deficient mice	(274, 275)
*IRF8*	Transcription factor that inhibits PC differentiation together with PU.1 and distribution into FO or MZ compartments	Increased expression in EBV-transformed cells	Increased IRF8 expression presumably would decrease PC differentiation	Increased function of IRF8 could lead to enhanced GC responses, through regulation of Bcl-6, AID, and MDM2	(276–278)
*IKZF3* (Aiolos)	Transcription factor involved in lymphocyte development and function; important in B cell maturation and activation	Unknown	Deficiency impairs the MZ B cell compartment but not the generation of short-lived PCs following immunization	Deficiency induces spontaneous GCs and production of autoantibodies; deficiency prevents generation of high-affinity BM PCs following immunization	(279–281)
*ETS1*	Transcription factor important for lymphocyte development and differentiation; it is also involved in maintaining B cell tolerance through anergy maintenance	Reduced expression of Ets1 in EBV-transformed cells and PBMCs	Increased EF PC responses in Ets1−/−	Loss of anergy may increase participation of autoreactive B cells in GC reactions	(282–285)
*FASL*	Apoptosis of immune cells through engagement of Fas	Increased surface expression on circulating fibrocytes	Based on studies in MRL/lpr mice, increased expression of FasL would be expected to decrease EF PC responses	Fas/FasL interactions are important for apoptosis in GC, thereby potentially affecting survival and selection of autoreactive GC B cells	(286, 287)
*TLR7*	Activation of immune cells by RNA viruses or self-antigen	Increased expression in PBMCs	EF PCs in AM14 Tg MRL/lpr mice are driven by TLR-7	GC responses are driven by B cell-intrinsic TLR7 in B6 and B6.Sle1b mice and FDCs in 564Igi RNP-specific lupus model	(184, 208, 210, 288, 289)
*SLC15A4*	Lysosomal amino-acid transporter required for endosomal TLR signaling and IFN1 production	One risk variant is associated with reduced expression in monocytes	SLC15a4 mutant mice, which are unable to produce IFN1, have reduced anti-chromatin IgG and IgM in B6/lpr mice, but have otherwise normal T-dependent and independent responses	T-dependent IgG2a/c OVA responses are decreased in SLC15a4−/− mice, but not in SLC15a4 mutant mice. IgG autoantibodies in pristane-induced lupus aC15a4 deficiency, suggesting that reduced expression in risk allele carriers may block GC-derived PCs	(290–293)

*The categories “potential role in EF or GC” are speculative and based on our understanding of the literature included in the reference column*.

Certain risk alleles, such as HLA class II genes, FCGR2B, STAT4, CD80, IRF8, and PRDM1, most likely drive GC responses, whereas other risk alleles, such as ETS1, LYN, BACH2, and BLK, may preferentially drive EF responses in SLE, although this pathway has not been extensively explored. Further understanding of the exact role of each risk allele in plasma cell differentiation pathways may enhance our insight into patient heterogeneity.

## Conclusion

In this review, we have described the PC differentiation pathways which can contribute to the development of autoantibody production in SLE. Whereas both EF and GC pathways may be active in the same patient, we propose that certain genetic risk alleles contribute to the dominance of one of these pathways. The dominant PC differentiation pathway, determined by the composite of risk alleles, may contribute to patient heterogeneity and to response to therapy. Although it is likely that different therapeutics alter each pathway to a different extent, there is to our knowledge not enough understanding of the molecular pathways in each response nor is there clear evidence which therapeutics target which pathway. These pathways are not as distinct as we thought, nor can the pathway taken by a PC be easily distinguished with current knowledge. A more thorough analysis of these pathways, their role in SLE, and the contribution of genetic risk alleles to each pathway may provide us with distinct targets to allow precision therapy.

## Author Contributions

SM, AB, and YA-F contributed to writing the manuscript. JS and BD contributed to the concept, reviewing, and writing of the manuscript.

## Conflict of Interest Statement

The authors declare that the research was conducted in the absence of any commercial or financial relationships that could be construed as a potential conflict of interest. The reviewer RM and handling editor declared their shared affiliation.

## Abbreviations

AID, activation-induced cytidine deaminase; BCR, B cell receptor; BM, bone marrow; CSR, class switch recombination; DC, dendritic cell; EF, extrafollicular; FDC, follicular dendritic cell; FO, follicular; GC, germinal center; ICOS, inducible T-cell costimulator; IFN, interferon; MHC, major histocompatibility complex; MZ, marginal zone; PB, plasmablast; PBMC, peripheral blood mononuclear cell; PC, plasma cell; pDC, plasmacytoid dendritic cell; SHM, somatic hypermutation; SLE, systemic lupus erythematosus; Tfh, T follicular helper; TLR, Toll-like receptor.

## References

[1] Suurmond J Diamond B. Autoantibodies in systemic autoimmune diseases: specificity and pathogenicity. J Clin Invest (2015) 125(6):2194–202. 10.1172/JCI78084 25938780 PMC4497746

[2] Tan EM Cohen AS Fries JF Masi AT McShane DJ Rothfield NF The 1982 revised criteria for the classification of systemic lupus erythematosus. Arthritis Rheum (1982) 25(11):1271–7. 10.1002/art.1780251101 7138600

[3] Vas J Gronwall C Marshak-Rothstein A Silverman GJ. Natural antibody to apoptotic cell membranes inhibits the proinflammatory properties of lupus autoantibody immune complexes. Arthritis Rheum (2012) 64(10):3388–98. 10.1002/art.34537 22577035 PMC3462267

[4] Li QZ Xie C Wu T Mackay M Aranow C Putterman C Identification of autoantibody clusters that best predict lupus disease activity using glomerular proteome arrays. J Clin Invest (2005) 115(12):3428–39. 10.1172/JCI23587 16322790 PMC1297234

[5] Mannoor K Matejuk A Xu Y Beardall M Chen C. Expression of natural autoantibodies in MRL-lpr mice protects from lupus nephritis and improves survival. J Immunol (2012) 188(8):3628–38. 10.4049/jimmunol.1102859 22407922

[6] Nemazee D Buerki K. Clonal deletion of autoreactive B lymphocytes in bone marrow chimeras. Proc Natl Acad Sci U S A (1989) 86(20):8039–43. 10.1073/pnas.86.20.8039 2682636 PMC298209

[7] Wardemann H Yurasov S Schaefer A Young JW Meffre E Nussenzweig MC. Predominant autoantibody production by early human B cell precursors. Science (2003) 301(5638):1374–7. 10.1126/science.1086907 12920303

[8] Pewzner-Jung Y Friedmann D Sonoda E Jung S Rajewsky K Eilat D B cell deletion, anergy, and receptor editing in “knock in” mice targeted with a germline-encoded or somatically mutated anti-DNA heavy chain. J Immunol (1998) 161(9):4634–45. 9794392

[9] Mandik-Nayak L Bui A Noorchashm H Eaton A Erikson J. Regulation of anti-double-stranded DNA B cells in nonautoimmune mice: localization to the T-B interface of the splenic follicle. J Exp Med (1997) 186(8):1257–67. 10.1084/jem.186.8.1257 9334365 PMC2199093

[10] Pugh-Bernard AE Silverman GJ Cappione AJ Villano ME Ryan DH Insel RA Regulation of inherently autoreactive VH4-34 B cells in the maintenance of human B cell tolerance. J Clin Invest (2001) 108(7):1061–70. 10.1172/JCI12462 11581307 PMC200949

[11] Nutt SL Hodgkin PD Tarlinton DM Corcoran LM. The generation of antibody-secreting plasma cells. Nat Rev Immunol (2015) 15(3):160–71. 10.1038/nri3795 25698678

[12] Chernova I Jones DD Wilmore JR Bortnick A Yucel M Hershberg U Lasting antibody responses are mediated by a combination of newly formed and established bone marrow plasma cells drawn from clonally distinct precursors. J Immunol (2014) 193(10):4971–9. 10.4049/jimmunol.1401264 25326027 PMC4234148

[13] Sanz I Wei C Lee FE Anolik J. Phenotypic and functional heterogeneity of human memory B cells. Semin Immunol (2008) 20(1):67–82. 10.1016/j.smim.2007.12.006 18258454 PMC2440717

[14] Kallies A Hasbold J Tarlinton DM Dietrich W Corcoran LM Hodgkin PD Plasma cell ontogeny defined by quantitative changes in blimp-1 expression. J Exp Med (2004) 200(8):967–77. 10.1084/jem.20040973 15492122 PMC2211847

[15] Jeurissen A Ceuppens JL Bossuyt X T lymphocyte dependence of the antibody response to ‘T lymphocyte independent type 2’ antigens. Immunology (2004) 111(1):1–7. 10.1111/j.1365-2567.2004.01775.x 14678191 PMC1782396

[16] Shih TA Roederer M Nussenzweig MC. Role of antigen receptor affinity in T cell-independent antibody responses in vivo. Nat Immunol (2002) 3(4):399–406. 10.1038/ni776 11896394

[17] Lentz VM Manser T. Cutting edge: germinal centers can be induced in the absence of T cells. J Immunol (2001) 167(1):15–20. 10.4049/jimmunol.167.1.15 11418626

[18] Mongini PK Stein KE Paul WE. T cell regulation of IgG subclass antibody production in response to T-independent antigens. J Exp Med (1981) 153(1):1–12. 10.1084/jem.153.1.1 6969777 PMC2186053

[19] Vinuesa CG Chang PP. Innate B cell helpers reveal novel types of antibody responses. Nat Immunol (2013) 14(2):119–26. 10.1038/ni.2511 23334833

[20] Khan AQ Chen Q Wu ZQ Paton JC Snapper CM. Both innate immunity and type 1 humoral immunity to *Streptococcus pneumoniae* are mediated by MyD88 but differ in their relative levels of dependence on toll-like receptor 2. Infect Immun (2005) 73(1):298–307. 10.1128/IAI.73.1.298-307.2005 15618166 PMC538967

[21] Hou B Saudan P Ott G Wheeler ML Ji M Kuzmich L Selective utilization of toll-like receptor and MyD88 signaling in B cells for enhancement of the antiviral germinal center response. Immunity (2011) 34(3):375–84. 10.1016/j.immuni.2011.01.011 21353603 PMC3064721

[22] Guay HM Andreyeva TA Garcea RL Welsh RM Szomolanyi-Tsuda E. MyD88 is required for the formation of long-term humoral immunity to virus infection. J Immunol (2007) 178(8):5124–31. 10.4049/jimmunol.178.8.5124 17404295

[23] Raval FM Mishra R Garcea RL Welsh RM Szomolanyi-Tsuda E. Long-lasting T cell-independent IgG responses require MyD88-mediated pathways and are maintained by high levels of virus persistence. MBio (2013) 4(6):e812–3. 10.1128/mBio.00812-13 24194540 PMC3892782

[24] Leadbetter EA Rifkin IR Hohlbaum AM Beaudette BC Shlomchik MJ Marshak-Rothstein A. Chromatin-IgG complexes activate B cells by dual engagement of IgM and toll-like receptors. Nature (2002) 416(6881):603–7. 10.1038/416603a 11948342

[25] Jego G Palucka AK Blanck JP Chalouni C Pascual V Banchereau J. Plasmacytoid dendritic cells induce plasma cell differentiation through type I interferon and interleukin 6. Immunity (2003) 19(2):225–34. 10.1016/S1074-7613(03)00208-5 12932356

[26] Litinskiy MB Nardelli B Hilbert DM He B Schaffer A Casali P DCs induce CD40-independent immunoglobulin class switching through BLyS and APRIL. Nat Immunol (2002) 3(9):822–9. 10.1038/ni829 12154359 PMC4621779

[27] Moore PA Belvedere O Orr A Pieri K LaFleur DW Feng P BLyS: member of the tumor necrosis factor family and B lymphocyte stimulator. Science (1999) 285(5425):260–3. 10.1126/science.285.5425.260 10398604

[28] He B Santamaria R Xu W Cols M Chen K Puga I The transmembrane activator TACI triggers immunoglobulin class switching by activating B cells through the adaptor MyD88. Nat Immunol (2010) 11(9):836–45. 10.1038/ni.1914 20676093 PMC3047500

[29] Stavnezer J Guikema JE Schrader CE. Mechanism and regulation of class switch recombination. Annu Rev Immunol (2008) 26:261–92. 10.1146/annurev.immunol.26.021607.090248 18370922 PMC2707252

[30] Rothaeusler K Baumgarth N. B-cell fate decisions following influenza virus infection. Eur J Immunol (2010) 40(2):366–77. 10.1002/eji.200939798 19946883 PMC2846450

[31] Puga I Cols M Barra CM He B Cassis L Gentile M B cell-helper neutrophils stimulate the diversification and production of immunoglobulin in the marginal zone of the spleen. Nat Immunol (2011) 13(2):170–80. 10.1038/ni.2194 22197976 PMC3262910

[32] Pone EJ Zhang J Mai T White CA Li G Sakakura JK BCR-signalling synergizes with TLR-signalling for induction of AID and immunoglobulin class-switching through the non-canonical NF-kappaB pathway. Nat Commun (2012) 3:767 10.1038/ncomms1769 22473011 PMC3337981

[33] Herzenberg LA Stall AM Lalor PA Sidman C Moore WA Parks DR The Ly-1 B cell lineage. Immunol Rev (1986) 93:81–102. 3096879 10.1111/j.1600-065x.1986.tb01503.x

[34] Hardy RR Carmack CE Li YS Hayakawa K. Distinctive developmental origins and specificities of murine CD5+ B cells. Immunol Rev (1994) 137:91–118. 7518415 10.1111/j.1600-065x.1994.tb00660.x

[35] Baumgarth N. The double life of a B-1 cell: self-reactivity selects for protective effector functions. Nat Rev Immunol (2011) 11(1):34–46. 10.1038/nri2901 21151033

[36] Hardy RR Hayakawa K CD5 B cells, a fetal B cell lineage. Adv Immunol (1994) 55:297–339. 7508175 10.1016/s0065-2776(08)60512-x

[37] Kasaian MT Casali P. Autoimmunity-prone B-1 (CD5 B) cells, natural antibodies and self recognition. Auto immunity (1993) 15(4):315–29. 10.3109/08916939309115755 7511005

[38] Hayakawa K Hardy RR Honda M Herzenberg LA Steinberg AD. Ly-1 B cells: functionally distinct lymphocytes that secrete IgM autoantibodies. Proc Natl Acad Sci U S A (1984) 81(8):2494–8. 10.1073/pnas.81.8.2494 6609363 PMC345088

[39] Griffin DO Holodick NE Rothstein TL Human B1 cells in umbilical cord and adult peripheral blood express the novel phenotype CD20+ CD27+ CD43+ CD70. J Exp Med (2011) 208(1):67–80. 10.1084/jem.20101499 21220451 PMC3023138

[40] Conger JD Sage HJ Corley RB. Correlation of antibody multireactivity with variable region primary structure among murine anti-erythrocyte autoantibodies. Eur J Immunol (1992) 22(3):783–90. 10.1002/eji.1830220323 1547822

[41] Sindhava VJ Bondada S. Multiple regulatory mechanisms control B-1 B cell activation. Front Immunol (2012) 3:372. 10.3389/fimmu.2012.00372 23251136 PMC3523257

[42] Cole LE Yang Y Elkins KL Fernandez ET Qureshi N Shlomchik MJ Antigen-specific B-1a antibodies induced by *Francisella tularensis* LPS provide long-term protection against *F. tularensis* LVS challenge. Proc Natl Acad Sci U S A (2009) 106(11):4343–8. 10.1073/pnas.0813411106 19251656 PMC2657382

[43] Berland R Wortis HH. Origins and functions of B-1 cells with notes on the role of CD5. Annu Rev Immunol (2002) 20:253–300. 10.1146/annurev.immunol.20.100301.064833 11861604

[44] Haas KM Poe JC Steeber DA Tedder TF B-1a and B-1b cells exhibit distinct developmental requirements and have unique functional roles in innate and adaptive immunity to S. pneumoniae. Immunity (2005) 23(1):7–18. 10.1016/j.immuni.2005.04.011 16039575

[45] Alugupalli KR Leong JM Woodland RT Muramatsu M Honjo T Gerstein RM. B1b lymphocytes confer T cell-independent long-lasting immunity. Immunity (2004) 21(3):379–90. 10.1016/j.immuni.2004.06.019 15357949

[46] Linton PJ Lo D Lai L Thorbecke GJ Klinman NR. Among naive precursor cell subpopulations only progenitors of memory B cells originate germinal centers. Eur J Immunol (1992) 22(5):1293–7. 1374340 10.1002/eji.1830220526

[47] Mantovani L Wilder RL Casali P. Human rheumatoid B-1a (CD5+ B) cells make somatically hypermutated high affinity IgM rheumatoid factors. J Immunol (1993) 151(1):473–88. 7686945 PMC4625548

[48] Murakami M Yoshioka H Shirai T Tsubata T Honjo T. Prevention of autoimmune symptoms in autoimmune-prone mice by elimination of B-1 cells. Int Immunol (1995) 7(5):877–82. 10.1093/intimm/7.5.877 7547714

[49] Casali P Notkins AL Probing the human B-cell repertoire with EBV: polyreactive antibodies and CD5+ B lymphocytes. Annu Rev Immunol (1989) 7:513–35. 10.1146/annurev.iy.07.040189.002501 2469441

[50] Suzuki N Sakane T Engleman EG. Anti-DNA antibody production by CD5+ and CD5- B cells of patients with systemic lupus erythematosus. J Clin Invest (1990) 85(1):238–47. 1688569 10.1172/JCI114418 PMC296411

[51] Martin F Oliver AM Kearney JF. Marginal zone and B1 B cells unite in the early response against T-independent blood-borne particulate antigens. Immunity (2001) 14(5):617–29. 10.1016/S1074-7613(01)00129-7 11371363

[52] Genestier L Taillardet M Mondiere P Gheit H Bella C Defrance T TLR agonists selectively promote terminal plasma cell differentiation of B cell subsets specialized in thymus-independent responses. J Immunol (2007) 178(12):7779–86. 10.4049/jimmunol.178.12.7779 17548615

[53] Oliver AM Martin F Gartland GL Carter RH Kearney JF. Marginal zone B cells exhibit unique activation, proliferative and immunoglobulin secretory responses. Eur J Immunol (1997) 27(9):2366–74. 10.1002/eji.1830270935 9341782

[54] Balazs M Martin F Zhou T Kearney J. Blood dendritic cells interact with splenic marginal zone B cells to initiate T-independent immune responses. Immunity (2002) 17(3):341–52. 10.1016/S1074-7613(02)00389-8 12354386

[55] Bialecki E Paget C Fontaine J Capron M Trottein F Faveeuw C. Role of marginal zone B lymphocytes in invariant NKT cell activation. J Immunol (2009) 182(10):6105–13. 10.4049/jimmunol.0802273 19414762

[56] van den Eertwegh AJ Laman JD Schellekens MM Boersma WJ Claassen E. Complement-mediated follicular localization of T-independent type-2 antigens: the role of marginal zone macrophages revisited. Eur J Immunol (1992) 22(3):719–26. 10.1002/eji.1830220315 1547818

[57] Liu YJ Zhang J Lane PJ Chan EY MacLennan IC. Sites of specific B cell activation in primary and secondary responses to T cell-dependent and T cell-independent antigens. Eur J Immunol (1991) 21(12):2951–62. 10.1002/eji.1830211209 1748148

[58] Pao LI Lam KP Henderson JM Kutok JL Alimzhanov M Nitschke L B cell-specific deletion of protein-tyrosine phosphatase Shp1 promotes B-1a cell development and causes systemic autoimmunity. Immunity (2007) 27(1):35–48. 10.1016/j.immuni.2007.04.016 17600736

[59] Groom JR Fletcher CA Walters SN Grey ST Watt SV Sweet MJ BAFF and MyD88 signals promote a lupuslike disease independent of T cells. J Exp Med (2007) 204(8):1959–71. 10.1084/jem.20062567 17664289 PMC2118661

[60] MacLennan IC Toellner KM Cunningham AF Serre K Sze DM Zuniga E Extrafollicular antibody responses. Immunol Rev (2003) 194:8–18. 10.1034/j.1600-065X.2003.00058.x 12846803

[61] Song H Cerny J. Functional heterogeneity of marginal zone B cells revealed by their ability to generate both early antibody-forming cells and germinal centers with hypermutation and memory in response to a T-dependent antigen. J Exp Med (2003) 198(12):1923–35. 10.1084/jem.20031498 14662910 PMC2194154

[62] Oliver AM Martin F Kearney JF. IgMhighCD21high lymphocytes enriched in the splenic marginal zone generate effector cells more rapidly than the bulk of follicular B cells. J Immunol (1999) 162(12):7198–207. 10358166

[63] Attanavanich K Kearney JF Marginal zone, but not follicular B cells, are potent activators of naive CD4 T cells. J Immunol (2004) 172(2):803–11. 10.4049/jimmunol.172.2.803 14707050

[64] Chappell CP Draves KE Giltiay NV Clark EA. Extrafollicular B cell activation by marginal zone dendritic cells drives T cell-dependent antibody responses. J Exp Med (2012) 209(10):1825–40. 10.1084/jem.20120774 22966002 PMC3457737

[65] Rubtsov A Strauch P Digiacomo A Hu J Pelanda R Torres RM. Lsc regulates marginal-zone B cell migration and adhesion and is required for the IgM T-dependent antibody response. Immunity (2005) 23(5):527–38. 10.1016/j.immuni.2005.09.018 16286020

[66] Cinamon G Zachariah MA Lam OM Foss FW Jr Cyster JG. Follicular shuttling of marginal zone B cells facilitates antigen transport. Nat Immunol (2008) 9(1):54–62. 10.1038/ni1542 18037889 PMC2488964

[67] Reif K Ekland EH Ohl L Nakano H Lipp M Forster R Balanced responsiveness to chemoattractants from adjacent zones determines B-cell position. Nature (2002) 416(6876):94–9. 10.1038/416094a 11882900

[68] Pereira JP Kelly LM Xu Y Cyster JG. EBI2 mediates B cell segregation between the outer and centre follicle. Nature (2009) 460(7259):1122–6. 10.1038/nature08226 19597478 PMC2809436

[69] Toellner KM Luther SA Sze DM Choy RK Taylor DR MacLennan IC T helper 1 (Th1) and Th2 characteristics start to develop during T cell priming and are associated with an immediate ability to induce immunoglobulin class switching. J Exp Med (1998) 187(8):1193–204. 10.1084/jem.187.8.1193 9547331 PMC2212236

[70] Rousset F Garcia E Defrance T Peronne C Vezzio N Hsu DH Interleukin 10 is a potent growth and differentiation factor for activated human B lymphocytes. Proc Natl Acad Sci U S A (1992) 89(5):1890–3. 10.1073/pnas.89.5.1890 1371884 PMC48559

[71] Lundgren M Persson U Larsson P Magnusson C Smith CI Hammarstrom L Interleukin 4 induces synthesis of IgE and IgG4 in human B cells. Eur J Immunol (1989) 19(7):1311–5. 10.1002/eji.1830190724 2788092

[72] Bryant VL Ma CS Avery DT Li Y Good KL Corcoran LM Cytokine-mediated regulation of human B cell differentiation into Ig-secreting cells: predominant role of IL-21 produced by CXCR5+ T follicular helper cells. J Immunol (2007) 179(12):8180–90. 10.4049/jimmunol.179.12.8180 18056361

[73] Avery DT Deenick EK Ma CS Suryani S Simpson N Chew GY B cell-intrinsic signaling through IL-21 receptor and STAT3 is required for establishing long-lived antibody responses in humans. J Exp Med (2010) 207(1):155–71. 10.1084/jem.20091706 20048285 PMC2812540

[74] Jacob J Kelsoe G In situ studies of the primary immune response to (4-hydroxy-3-nitrophenyl)acetyl. II. A common clonal origin for periarteriolar lymphoid sheath-associated foci and germinal centers. J Exp Med (1992) 176(3):679–87. 10.1084/jem.176.3.679 1512536 PMC2119370

[75] Chan TD Gatto D Wood K Camidge T Basten A Brink R. Antigen affinity controls rapid T-dependent antibody production by driving the expansion rather than the differentiation or extrafollicular migration of early plasmablasts. J Immunol (2009) 183(5):3139–49. 10.4049/jimmunol.0901690 19666691

[76] Phan TG Paus D Chan TD Turner ML Nutt SL Basten A High affinity germinal center B cells are actively selected into the plasma cell compartment. J Exp Med (2006) 203(11):2419–24. 10.1084/jem.20061254 17030950 PMC2118125

[77] Schwickert TA Victora GD Fooksman DR Kamphorst AO Mugnier MR Gitlin AD A dynamic T cell-limited checkpoint regulates affinity-dependent B cell entry into the germinal center. J Exp Med (2011) 208(6):1243–52. 10.1084/jem.20102477 21576382 PMC3173244

[78] Rookhuizen DC DeFranco AL. Toll-like receptor 9 signaling acts on multiple elements of the germinal center to enhance antibody responses. Proc Natl Acad Sci U S A (2014) 111(31):E3224–33. 10.1073/pnas.1323985111 25053813 PMC4128120

[79] Garcia De Vinuesa C Gulbranson-Judge A Khan M O’Leary P Cascalho M Wabl M Dendritic cells associated with plasmablast survival. Eur J Immunol (1999) 29(11):3712–21. 10.1002/(SICI)1521-4141(199911)29:11<3712::AID-IMMU3712>3.0.CO;2-P 10556827

[80] Lee SK Rigby RJ Zotos D Tsai LM Kawamoto S Marshall JL B cell priming for extrafollicular antibody responses requires Bcl-6 expression by T cells. J Exp Med (2011) 208(7):1377–88. 10.1084/jem.20102065 21708925 PMC3135363

[81] Blink EJ Light A Kallies A Nutt SL Hodgkin PD Tarlinton DM Early appearance of germinal center-derived memory B cells and plasma cells in blood after primary immunization. J Exp Med (2005) 201(4):545 10.1084/jem.20042060 15710653 PMC2213050

[82] Toyama H Okada S Hatano M Takahashi Y Takeda N Ichii H Memory B cells without somatic hypermutation are generated from Bcl6-deficient B cells. Immunity (2002) 17(3):329–39. 10.1016/S1074-7613(02)00387-4 12354385

[83] Di Niro R Lee SJ Vander Heiden JA Elsner RA Trivedi N Bannock JM Salmonella infection drives promiscuous B cell activation followed by extrafollicular affinity maturation. Immunity (2015) 43(1):120–31. 10.1016/j.immuni.2015.06.013 26187411 PMC4523395

[84] Wang X Cho B Suzuki K Xu Y Green JA An J Follicular dendritic cells help establish follicle identity and promote B cell retention in germinal centers. J Exp Med (2011) 208(12):2497–510. 10.1084/jem.20111449 22042977 PMC3256970

[85] Heesters BA Chatterjee P Kim YA Gonzalez SF Kuligowski MP Kirchhausen T Endocytosis and recycling of immune complexes by follicular dendritic cells enhances B cell antigen binding and activation. Immunity (2013) 38(6):1164–75. 10.1016/j.immuni.2013.02.023 23770227 PMC3773956

[86] Gatto D Brink R The germinal center reaction. J Allergy Clin Immunol (2010) 126(5):898–907. 10.1016/j.jaci.2010.09.007 21050940

[87] Legler DF Loetscher M Roos RS Clark-Lewis I Baggiolini M Moser B B cell-attracting chemokine 1, a human CXC chemokine expressed in lymphoid tissues, selectively attracts B lymphocytes via BLR1/CXCR5. J Exp Med (1998) 187(4):655–60. 10.1084/jem.187.4.655 9463416 PMC2212150

[88] Allen CD Okada T Tang HL Cyster JG. Imaging of germinal center selection events during affinity maturation. Science (2007) 315(5811):528–31. 10.1126/science.1136736 17185562

[89] Qi H Cannons JL Klauschen F Schwartzberg PL Germain RN. SAP-controlled T-B cell interactions underlie germinal centre formation. Nature (2008) 455(7214):764–9. 10.1038/nature07345 18843362 PMC2652134

[90] Cortini A Ellinghaus U Malik TH Cunninghame Graham DS Botto M Vyse TJ B cell OX40L supports T follicular helper cell development and contributes to SLE pathogenesis. Ann Rheum Dis (2017) 76(12):2095–103. 10.1136/annrheumdis-2017-211499 28818832 PMC5705841

[91] Good-Jacobson KL Song E Anderson S Sharpe AH Shlomchik MJ. CD80 expression on B cells regulates murine T follicular helper development, germinal center B cell survival, and plasma cell generation. J Immunol (2012) 188(9):4217–25. 10.4049/jimmunol.1102885 22450810 PMC3331930

[92] Kitano M Moriyama S Ando Y Hikida M Mori Y Kurosaki T Bcl6 protein expression shapes pre-germinal center B cell dynamics and follicular helper T cell heterogeneity. Immunity (2011) 34(6):961–72. 10.1016/j.immuni.2011.03.025 21636294

[93] Noelle RJ Ledbetter JA Aruffo A. CD40 and its ligand, an essential ligand-receptor pair for thymus-dependent B-cell activation. Immunol Today (1992) 13(11):431–3. 1282319 10.1016/0167-5699(92)90068-I

[94] Allen RC Armitage RJ Conley ME Rosenblatt H Jenkins NA Copeland NG CD40 ligand gene defects responsible for X-linked hyper-IgM syndrome. Science (1993) 259(5097):990–3. 10.1126/science.7679801 7679801

[95] Taylor JJ Pape KA Jenkins MK A germinal center-independent pathway generates unswitched memory B cells early in the primary response. J Exp Med (2012) 209(3):597–606. 10.1084/jem.20111696 22370719 PMC3302224

[96] Linterman MA Beaton L Yu D Ramiscal RR Srivastava M Hogan JJ IL-21 acts directly on B cells to regulate Bcl-6 expression and germinal center responses. J Exp Med (2010) 207(2):353–63. 10.1084/jem.20091738 20142429 PMC2822609

[97] El Shikh ME El Sayed RM Sukumar S Szakal AK Tew JG. Activation of B cells by antigens on follicular dendritic cells. Trends Immunol (2010) 31(6):205–11. 10.1016/j.it.2010.03.002 20418164 PMC2886728

[98] Ersching J Efeyan A Mesin L Jacobsen JT Pasqual G Grabiner BC Germinal center selection and affinity maturation require dynamic regulation of mTORC1 kinase. Immunity (2017) 46(6):1045–58e6. 10.1016/j.immuni.2017.06.005 28636954 PMC5526448

[99] Gitlin AD Shulman Z Nussenzweig MC. Clonal selection in the germinal centre by regulated proliferation and hypermutation. Nature (2014) 509(7502):637–40. 10.1038/nature13300 24805232 PMC4271732

[100] Victora GD Schwickert TA Fooksman DR Kamphorst AO Meyer-Hermann M Dustin ML Germinal center dynamics revealed by multiphoton microscopy with a photoactivatable fluorescent reporter. Cell (2010) 143(4):592–605. 10.1016/j.cell.2010.10.032 21074050 PMC3035939

[101] Klein U Dalla-Favera R. Germinal centres: role in B-cell physiology and malignancy. Nat Rev Immunol (2008) 8(1):22–33. 10.1038/nri2217 18097447

[102] Hao Z Duncan GS Seagal J Su YW Hong C Haight J Fas receptor expression in germinal-center B cells is essential for T and B lymphocyte homeostasis. Immunity (2008) 29(4):615–27. 10.1016/j.immuni.2008.07.016 18835195 PMC3470429

[103] Chan TD Wood K Hermes JR Butt D Jolly CJ Basten A Elimination of germinal-center-derived self-reactive B cells is governed by the location and concentration of self-antigen. Immunity (2012) 37(5):893–904. 10.1016/j.immuni.2012.07.017 23142780

[104] Mayer CT Gazumyan A Kara EE Gitlin AD Golijanin J Viant C The microanatomic segregation of selection by apoptosis in the germinal center. Science (2017) 358(6360):eaao2602 10.1126/science.aao2602 28935768 PMC5957278

[105] Shinnakasu R Inoue T Kometani K Moriyama S Adachi Y Nakayama M Regulated selection of germinal-center cells into the memory B cell compartment. Nat Immunol (2016) 17(7):861–9. 10.1038/ni.3460 27158841

[106] Smith KG Light A O’Reilly LA Ang SM Strasser A Tarlinton D. Bcl-2 transgene expression inhibits apoptosis in the germinal center and reveals differences in the selection of memory B cells and bone marrow antibody-forming cells. J Exp Med (2000) 191(3):475–84. 10.1084/jem.191.3.475 10662793 PMC2195819

[107] Smith KG Light A Nossal GJ Tarlinton DM. The extent of affinity maturation differs between the memory and antibody-forming cell compartments in the primary immune response. EMBO J (1997) 16(11):2996–3006. 10.1093/emboj/16.11.2996 9214617 PMC1169918

[108] Krautler NJ Suan D Butt D Bourne K Hermes JR Chan TD Differentiation of germinal center B cells into plasma cells is initiated by high-affinity antigen and completed by Tfh cells. J Exp Med (2017) 214(5):1259–67. 10.1084/jem.20161533 28363897 PMC5413338

[109] Weisel FJ Zuccarino-Catania GV Chikina M Shlomchik MJ A temporal switch in the germinal center determines differential output of memory B and plasma cells. Immunity (2016) 44(1):116–30. 10.1016/j.immuni.2015.12.004 26795247 PMC4724390

[110] Zotos D Coquet JM Zhang Y Light A D’Costa K Kallies A IL-21 regulates germinal center B cell differentiation and proliferation through a B cell-intrinsic mechanism. J Exp Med (2010) 207(2):365–78. 10.1084/jem.20091777 20142430 PMC2822601

[111] Moens L Tangye SG. Cytokine-mediated regulation of plasma cell generation: IL-21 takes center stage. Front Immunol (2014) 5:65. 10.3389/fimmu.2014.00065 24600453 PMC3927127

[112] Kasturi SP Skountzou I Albrecht RA Koutsonanos D Hua T Nakaya HI Programming the magnitude and persistence of antibody responses with innate immunity. Nature (2011) 470(7335):543–7. 10.1038/nature09737 21350488 PMC3057367

[113] Gavin AL Hoebe K Duong B Ota T Martin C Beutler B Adjuvant-enhanced antibody responses in the absence of toll-like receptor signaling. Science (2006) 314(5807):1936–8. 10.1126/science.1135299 17185603 PMC1868398

[114] Meyer-Bahlburg A Khim S Rawlings DJ B cell intrinsic TLR signals amplify but are not required for humoral immunity. J Exp Med (2007) 204(13):3095–101. 10.1084/jem.20071250 18039950 PMC2150979

[115] Suan D Sundling C Brink R. Plasma cell and memory B cell differentiation from the germinal center. Curr Opin Immunol (2017) 45:97–102. 10.1016/j.coi.2017.03.006 28319733

[116] Chiu YK Lin IY Su ST Wang KH Yang SY Tsai DY Transcription factor ABF-1 suppresses plasma cell differentiation but facilitates memory B cell formation. J Immunol (2014) 193(5):2207–17. 10.4049/jimmunol.1400411 25070843

[117] Reimold AM Iwakoshi NN Manis J Vallabhajosyula P Szomolanyi-Tsuda E Gravallese EM Plasma cell differentiation requires the transcription factor XBP-1. Nature (2001) 412(6844):300–7. 10.1038/35085509 11460154

[118] Minnich M Tagoh H Bonelt P Axelsson E Fischer M Cebolla B Multifunctional role of the transcription factor Blimp-1 in coordinating plasma cell differentiation. Nat Immunol (2016) 17(3):331–43. 10.1038/ni.3349 26779602 PMC5790184

[119] Sciammas R Shaffer AL Schatz JH Zhao H Staudt LM Singh H. Graded expression of interferon regulatory factor-4 coordinates isotype switching with plasma cell differentiation. Immunity (2006) 25(2):225–36. 10.1016/j.immuni.2006.07.009 16919487

[120] Nera KP Kohonen P Narvi E Peippo A Mustonen L Terho P Loss of Pax5 promotes plasma cell differentiation. Immunity (2006) 24(3):283–93. 10.1016/j.immuni.2006.02.003 16546097

[121] Shaffer AL Lin KI Kuo TC Yu X Hurt EM Rosenwald A Blimp-1 orchestrates plasma cell differentiation by extinguishing the mature B cell gene expression program. Immunity (2002) 17(1):51–62. 10.1016/S1074-7613(02)00335-7 12150891

[122] Shaffer AL Shapiro-Shelef M Iwakoshi NN Lee AH Qian SB Zhao H XBP1, downstream of Blimp-1, expands the secretory apparatus and other organelles, and increases protein synthesis in plasma cell differentiation. Immunity (2004) 21(1):81–93. 10.1016/j.immuni.2004.06.010 15345222

[123] Meyer-Hermann M Mohr E Pelletier N Zhang Y Victora GD Toellner KM A theory of germinal center B cell selection, division, and exit. Cell Rep (2012) 2(1):162–74. 10.1016/j.celrep.2012.05.010 22840406

[124] Mohr E Serre K Manz RA Cunningham AF Khan M Hardie DL Dendritic cells and monocyte/macrophages that create the IL-6/APRIL-rich lymph node microenvironments where plasmablasts mature. J Immunol (2009) 182(4):2113–23. 10.4049/jimmunol.0802771 19201864

[125] Tipton CM Fucile CF Darce J Chida A Ichikawa T Gregoretti I Diversity, cellular origin and autoreactivity of antibody-secreting cell population expansions in acute systemic lupus erythematosus. Nat Immunol (2015) 16(7):755–65. 10.1038/ni.3175 26006014 PMC4512288

[126] Slifka MK Antia R Whitmire JK Ahmed R. Humoral immunity due to long-lived plasma cells. Immunity (1998) 8(3):363–72. 10.1016/S1074-7613(00)80541-5 9529153

[127] Manz RA Thiel A Radbruch A Lifetime of plasma cells in the bone marrow. Nature (1997) 388(6638):133–4. 10.1038/40540 9217150

[128] Hammarlund E Thomas A Amanna IJ Holden LA Slayden OD Park B Plasma cell survival in the absence of B cell memory. Nat Commun (2017) 8(1):1781. 10.1038/s41467-017-01901-w 29176567 PMC5701209

[129] Bhoj VG Arhontoulis D Wertheim G Capobianchi J Callahan CA Ellebrecht CT Persistence of long-lived plasma cells and humoral immunity in individuals responding to CD19-directed CAR T-cell therapy. Blood (2016) 128(3):360–70. 10.1182/blood-2016-01-694356 27166358 PMC4957161

[130] Hiepe F Radbruch A. Plasma cells as an innovative target in autoimmune disease with renal manifestations. Nat Rev Nephrol (2016) 12(4):232–40. 10.1038/nrneph.2016.20 26923204

[131] Taddeo A Khodadadi L Voigt C Mumtaz IM Cheng Q Moser K Long-lived plasma cells are early and constantly generated in New Zealand Black/New Zealand White F1 mice and their therapeutic depletion requires a combined targeting of autoreactive plasma cells and their precursors. Arthritis Res Ther (2015) 17:39. 10.1186/s13075-015-0551-3 25889236 PMC4411657

[132] Sze DM Toellner KM Garcia de Vinuesa C Taylor DR MacLennan IC Intrinsic constraint on plasmablast growth and extrinsic limits of plasma cell survival. J Exp Med (2000) 192(6):813–21. 10.1084/jem.192.6.813 10993912 PMC2193289

[133] Minges Wols HA Ippolito JA Yu Z Palmer JL White FA Le PT The effects of microenvironment and internal programming on plasma cell survival. Int Immunol (2007) 19(7):837–46. 10.1093/intimm/dxm051 17606982

[134] Kometani K Kurosaki T. Differentiation and maintenance of long-lived plasma cells. Curr Opin Immunol (2015) 33:64–9. 10.1016/j.coi.2015.01.017 25677584

[135] Tarte K Zhan F De Vos J Klein B Shaughnessy J Jr. Gene expression profiling of plasma cells and plasmablasts: toward a better understanding of the late stages of B-cell differentiation. Blood (2003) 102(2):592–600. 10.1182/blood-2002-10-3161 12663452

[136] Tokoyoda K Egawa T Sugiyama T Choi BI Nagasawa T. Cellular niches controlling B lymphocyte behavior within bone marrow during development. Immunity (2004) 20(6):707–18. 10.1016/j.immuni.2004.05.001 15189736

[137] Chu VT Frohlich A Steinhauser G Scheel T Roch T Fillatreau S Eosinophils are required for the maintenance of plasma cells in the bone marrow. Nat Immunol (2011) 12(2):151–9. 10.1038/ni.1981 21217761

[138] Wilmore JR Allman D Here, there, and anywhere? Arguments for and against the physical plasma cell survival niche. J Immunol (2017) 199(3):839–45. 10.4049/jimmunol.1700461 28739594 PMC5651088

[139] Belnoue E Pihlgren M McGaha TL Tougne C Rochat AF Bossen C APRIL is critical for plasmablast survival in the bone marrow and poorly expressed by early-life bone marrow stromal cells. Blood (2008) 111(5):2755–64. 10.1182/blood-2007-09-110858 18180376

[140] Benson MJ Dillon SR Castigli E Geha RS Xu S Lam KP Cutting edge: the dependence of plasma cells and independence of memory B cells on BAFF and APRIL. J Immunol (2008) 180(6):3655–9. 10.4049/jimmunol.180.6.3655 18322170

[141] O’Connor BP Raman VS Erickson LD Cook WJ Weaver LK Ahonen C BCMA is essential for the survival of long-lived bone marrow plasma cells. J Exp Med (2004) 199(1):91–8. 10.1084/jem.20031330 14707116 PMC1887725

[142] Mahevas M Michel M Weill JC Reynaud CA. Long-lived plasma cells in autoimmunity: lessons from B-cell depleting therapy. Front Immunol (2013) 4:494. 10.3389/fimmu.2013.00494 24409184 PMC3873528

[143] Mahevas M Patin P Huetz F Descatoire M Cagnard N Bole-Feysot C B cell depletion in immune thrombocytopenia reveals splenic long-lived plasma cells. J Clin Invest (2013) 123(1):432–42. 10.1172/JCI65689 23241960 PMC3533302

[144] Rozanski CH Arens R Carlson LM Nair J Boise LH Chanan-Khan AA Sustained antibody responses depend on CD28 function in bone marrow-resident plasma cells. J Exp Med (2011) 208(7):1435–46. 10.1084/jem.20110040 21690252 PMC3135367

[145] Chevrier S Genton C Kallies A Karnowski A Otten LA Malissen B CD93 is required for maintenance of antibody secretion and persistence of plasma cells in the bone marrow niche. Proc Natl Acad Sci U S A (2009) 106(10):3895–900. 10.1073/pnas.0809736106 19228948 PMC2656176

[146] Hsu MC Toellner KM Vinuesa CG Maclennan IC B cell clones that sustain long-term plasmablast growth in T-independent extrafollicular antibody responses. Proc Natl Acad Sci U S A (2006) 103(15):5905–10. 10.1073/pnas.0601502103 16585532 PMC1424660

[147] Bortnick A Chernova I Quinn WJ III Mugnier M Cancro MP Allman D. Long-lived bone marrow plasma cells are induced early in response to T cell-independent or T cell-dependent antigens. J Immunol (2012) 188(11):5389–96. 10.4049/jimmunol.1102808 22529295 PMC4341991

[148] Sanderson NS Zimmermann M Eilinger L Gubser C Schaeren-Wiemers N Lindberg RL Cocapture of cognate and bystander antigens can activate autoreactive B cells. Proc Natl Acad Sci U S A (2017) 114(4):734–9. 10.1073/pnas.1614472114 28057865 PMC5278454

[149] Son M Santiago-Schwarz F Al-Abed Y Diamond B. C1q limits dendritic cell differentiation and activation by engaging LAIR-1. Proc Natl Acad Sci U S A (2012) 109(46):E3160–7. 10.1073/pnas.1212753109 23093673 PMC3503216

[150] Hess C Winkler A Lorenz AK Holecska V Blanchard V Eiglmeier S T cell-independent B cell activation induces immunosuppressive sialylated IgG antibodies. J Clin Invest (2013) 123(9):3788–96. 10.1172/JCI65938 23979161 PMC3754242

[151] Pfeifle R Rothe T Ipseiz N Scherer HU Culemann S Harre U Regulation of autoantibody activity by the IL-23-TH17 axis determines the onset of autoimmune disease. Nat Immunol (2017) 18(1):104–13. 10.1038/ni.3579 27820809 PMC5164937

[152] Klinman NR The “clonal selection hypothesis” and current concepts of B cell tolerance. Immunity (1996) 5(3):189–95. 10.1016/S1074-7613(00)80314-3 8808674

[153] Linton PJ Rudie A Klinman NR. Tolerance susceptibility of newly generating memory B cells. J Immunol (1991) 146(12):4099–104. 2040792

[154] Malkiel S Jeganathan V Wolfson S Manjarrez Orduno N Marasco E Aranow C Checkpoints for autoreactive B cells in the peripheral blood of lupus patients assessed by flow cytometry. Arthritis Rheumatol (2016) 68(9):2210–20. 10.1002/art.39710 27059652 PMC5523861

[155] Cappione A III Anolik JH Pugh-Bernard A Barnard J Dutcher P Silverman G Germinal center exclusion of autoreactive B cells is defective in human systemic lupus erythematosus. J Clin Invest (2005) 115(11):3205–16. 10.1172/JCI24179 16211091 PMC1242189

[156] Sabouri Z Schofield P Horikawa K Spierings E Kipling D Randall KL Redemption of autoantibodies on anergic B cells by variable-region glycosylation and mutation away from self-reactivity. Proc Natl Acad Sci U S A (2014) 111(25):E2567–75. 10.1073/pnas.1406974111 24821781 PMC4078846

[157] Reed JH Jackson J Christ D Goodnow CC. Clonal redemption of autoantibodies by somatic hypermutation away from self-reactivity during human immunization. J Exp Med (2016) 213(7):1255–65. 10.1084/jem.20151978 27298445 PMC4925023

[158] Ait-Azzouzene D Kono DH Gonzalez-Quintial R McHeyzer-Williams LJ Lim M Wickramarachchi D Deletion of IgG-switched autoreactive B cells and defects in Fas(lpr) lupus mice. J Immunol (2010) 185(2):1015–27. 10.4049/jimmunol.1000698 20554953 PMC3641794

[159] Rice JS Newman J Wang C Michael DJ Diamond B. Receptor editing in peripheral B cell tolerance. Proc Natl Acad Sci U S A (2005) 102(5):1608–13. 10.1073/pnas.0409217102 15659547 PMC547880

[160] Hande S Notidis E Manser T. Bcl-2 obstructs negative selection of autoreactive, hypermutated antibody V regions during memory B cell development. Immunity (1998) 8(2):189–98. 10.1016/S1074-7613(00)80471-9 9492000

[161] Notidis E Heltemes L Manser T. Dominant, hierarchical induction of peripheral tolerance during foreign antigen-driven B cell development. Immunity (2002) 17(3):317–27. 10.1016/S1074-7613(02)00392-8 12354384

[162] Pulendran B Kannourakis G Nouri S Smith KG Nossal GJ. Soluble antigen can cause enhanced apoptosis of germinal-centre B cells. Nature (1995) 375(6529):331–4. 10.1038/375331a0 7753199

[163] Wang YH Diamond B B cell receptor revision diminishes the autoreactive B cell response after antigen activation in mice. J Clin Invest (2008) 118(8):2896–907. 10.1172/JCI35618 18636122 PMC2467385

[164] Scheid JF Mouquet H Kofer J Yurasov S Nussenzweig MC Wardemann H. Differential regulation of self-reactivity discriminates between IgG+ human circulating memory B cells and bone marrow plasma cells. Proc Natl Acad Sci U S A (2011) 108(44):18044–8. 10.1073/pnas.1113395108 22025722 PMC3207704

[165] Pelletier N McHeyzer-Williams LJ Wong KA Urich E Fazilleau N McHeyzer-Williams MG. Plasma cells negatively regulate the follicular helper T cell program. Nat Immunol (2010) 11(12):1110–8. 10.1038/ni.1954 21037578 PMC3058870

[166] William J Euler C Christensen S Shlomchik MJ. Evolution of autoantibody responses via somatic hypermutation outside of germinal centers. Science (2002) 297(5589):2066–70. 10.1126/science.1073924 12242446

[167] Deng R Hurtz C Song Q Yue C Xiao G Yu H Extrafollicular CD4+ T-B interactions are sufficient for inducing autoimmune-like chronic graft-versus-host disease. Nat Commun (2017) 8(1):978. 10.1038/s41467-017-00880-2 29042531 PMC5645449

[168] Odegard JM Marks BR DiPlacido LD Poholek AC Kono DH Dong C ICOS-dependent extrafollicular helper T cells elicit IgG production via IL-21 in systemic autoimmunity. J Exp Med (2008) 205(12):2873–86. 10.1084/jem.20080840 18981236 PMC2585848

[169] Luzina IG Atamas SP Storrer CE daSilva LC Kelsoe G Papadimitriou JC Spontaneous formation of germinal centers in autoimmune mice. J Leukoc Biol (2001) 70(4):578–84. 10.1189/jlb.70.4.578 11590194

[170] Li Y Li H Weigert M. Autoreactive B cells in the marginal zone that express dual receptors. J Exp Med (2002) 195(2):181–8. 10.1084/jem.20011453 11805145 PMC2193605

[171] Julien S Soulas P Garaud JC Martin T Pasquali JL B cell positive selection by soluble self-antigen. J Immunol (2002) 169(8):4198–204. 10.4049/jimmunol.169.8.4198 12370349

[172] Cariappa A Tang M Parng C Nebelitskiy E Carroll M Georgopoulos K The follicular versus marginal zone B lymphocyte cell fate decision is regulated by Aiolos, Btk, and CD21. Immunity (2001) 14(5):603–15. 10.1016/S1074-7613(01)00135-2 11371362

[173] Martin F Kearney JF. Positive selection from newly formed to marginal zone B cells depends on the rate of clonal production, CD19, and btk. Immunity (2000) 12(1):39–49. 10.1016/S1074-7613(00)80157-0 10661404

[174] Cerutti A Cols M Puga I. Marginal zone B cells: virtues of innate-like antibody-producing lymphocytes. Nat Rev Immunol (2013) 13(2):118–32. 10.1038/nri3383 23348416 PMC3652659

[175] Phan TG Gardam S Basten A Brink R. Altered migration, recruitment, and somatic hypermutation in the early response of marginal zone B cells to T cell-dependent antigen. J Immunol (2005) 174(8):4567–78. 10.4049/jimmunol.174.8.4567 15814678

[176] Zhou Z Niu H Zheng YY Morel L. Autoreactive marginal zone B cells enter the follicles and interact with CD4+ T cells in lupus-prone mice. BMC Immunol (2011) 12:7. 10.1186/1471-2172-12-7 21251257 PMC3034709

[177] Grimaldi CM Michael DJ Diamond B. Cutting edge: expansion and activation of a population of autoreactive marginal zone B cells in a model of estrogen-induced lupus. J Immunol (2001) 167(4):1886–90. 10.4049/jimmunol.167.4.1886 11489967

[178] Wither JE Loh C Lajoie G Heinrichs S Cai YC Bonventi G Colocalization of expansion of the splenic marginal zone population with abnormal B cell activation and autoantibody production in B6 mice with an introgressed New Zealand Black chromosome 13 interval. J Immunol (2005) 175(7):4309–19. 10.4049/jimmunol.175.7.4309 16177071

[179] Atencio S Amano H Izui S Kotzin BL. Separation of the New Zealand Black genetic contribution to lupus from New Zealand Black determined expansions of marginal zone B and B1a cells. J Immunol (2004) 172(7):4159–66. 10.4049/jimmunol.172.7.4159 15034028

[180] Amano H Amano E Moll T Marinkovic D Ibnou-Zekri N Martinez-Soria E The Yaa mutation promoting murine lupus causes defective development of marginal zone B cells. J Immunol (2003) 170(5):2293–301. 10.4049/jimmunol.170.5.2293 12594250

[181] Nickerson KM Christensen SR Shupe J Kashgarian M Kim D Elkon K TLR9 regulates TLR7- and MyD88-dependent autoantibody production and disease in a murine model of lupus. J Immunol (2010) 184(4):1840–8. 10.4049/jimmunol.0902592 20089701 PMC4098568

[182] Teichmann LL Schenten D Medzhitov R Kashgarian M Shlomchik MJ. Signals via the adaptor MyD88 in B cells and DCs make distinct and synergistic contributions to immune activation and tissue damage in lupus. Immunity (2013) 38(3):528–40. 10.1016/j.immuni.2012.11.017 23499488 PMC3638041

[183] Christensen SR Kashgarian M Alexopoulou L Flavell RA Akira S Shlomchik MJ. Toll-like receptor 9 controls anti-DNA autoantibody production in murine lupus. J Exp Med (2005) 202(2):321–31. 10.1084/jem.20050338 16027240 PMC2212997

[184] Christensen SR Shupe J Nickerson K Kashgarian M Flavell RA Shlomchik MJ. Toll-like receptor 7 and TLR9 dictate autoantibody specificity and have opposing inflammatory and regulatory roles in a murine model of lupus. Immunity (2006) 25(3):417–28. 10.1016/j.immuni.2006.07.013 16973389

[185] Teichmann LL Ols ML Kashgarian M Reizis B Kaplan DH Shlomchik MJ. Dendritic cells in lupus are not required for activation of T and B cells but promote their expansion, resulting in tissue damage. Immunity (2010) 33(6):967–78. 10.1016/j.immuni.2010.11.025 21167752 PMC3010763

[186] Wong CK Wong PT Tam LS Li EK Chen DP Lam CW. Activation profile of toll-like receptors of peripheral blood lymphocytes in patients with systemic lupus erythematosus. Clin Exp Immunol (2010) 159(1):11–22. 10.1111/j.1365-2249.2009.04036.x 19843090 PMC2802691

[187] Flores-Borja F Kabouridis PS Jury EC Isenberg DA Mageed RA. Decreased Lyn expression and translocation to lipid raft signaling domains in B lymphocytes from patients with systemic lupus erythematosus. Arthritis Rheum (2005) 52(12):3955–65. 10.1002/art.21416 16320343

[188] Pritchard NR Cutler AJ Uribe S Chadban SJ Morley BJ Smith KG. Autoimmune-prone mice share a promoter haplotype associated with reduced expression and function of the Fc receptor FcgammaRII. Curr Biol (2000) 10(4):227–30. 10.1016/S0960-9822(00)00344-4 10704418

[189] Suurmond J Calise J Malkiel S Diamond B DNA-reactive B cells in lupus. Curr Opin Immunol (2016) 43:1–7. 10.1016/j.coi.2016.07.002 27504587 PMC5125853

[190] Chaturvedi A Dorward D Pierce SK. The B cell receptor governs the subcellular location of toll-like receptor 9 leading to hyperresponses to DNA-containing antigens. Immunity (2008) 28(6):799–809. 10.1016/j.immuni.2008.03.019 18513998 PMC2601674

[191] Lau CM Broughton C Tabor AS Akira S Flavell RA Mamula MJ RNA-associated autoantigens activate B cells by combined B cell antigen receptor/toll-like receptor 7 engagement. J Exp Med (2005) 202(9):1171–7. 10.1084/jem.20050630 16260486 PMC2213226

[192] Poovassery JS Bishop GA. Type I IFN receptor and the B cell antigen receptor regulate TLR7 responses via distinct molecular mechanisms. J Immunol (2012) 189(4):1757–64. 10.4049/jimmunol.1200624 22786773

[193] Boes M Schmidt T Linkemann K Beaudette BC Marshak-Rothstein A Chen J. Accelerated development of IgG autoantibodies and autoimmune disease in the absence of secreted IgM. Proc Natl Acad Sci U S A (2000) 97(3):1184–9. 10.1073/pnas.97.3.1184 10655505 PMC15562

[194] Haynes NM Allen CD Lesley R Ansel KM Killeen N Cyster JG. Role of CXCR5 and CCR7 in follicular Th cell positioning and appearance of a programmed cell death gene-1high germinal center-associated subpopulation. J Immunol (2007) 179(8):5099–108. 10.4049/jimmunol.179.8.5099 17911595

[195] Elsner RA Ernst DN Baumgarth N. Single and coexpression of CXCR4 and CXCR5 identifies CD4 T helper cells in distinct lymph node niches during influenza virus infection. J Virol (2012) 86(13):7146–57. 10.1128/JVI.06904-11 22532671 PMC3416343

[196] Herlands RA Christensen SR Sweet RA Hershberg U Shlomchik MJ T cell-independent and toll-like receptor-dependent antigen-driven activation of autoreactive B cells. Immunity (2008) 29(2):249–60. 10.1016/j.immuni.2008.06.009 18691914 PMC4106705

[197] Wang L Zhao P Ma L Shan Y Jiang Z Wang J Increased interleukin 21 and follicular helper T-like cells and reduced interleukin 10+ B cells in patients with new-onset systemic lupus erythematosus. J Rheumatol (2014) 41(9):1781–92. 10.3899/jrheum.131025 25028374

[198] He B Qiao X Cerutti A. CpG DNA induces IgG class switch DNA recombination by activating human B cells through an innate pathway that requires TLR9 and cooperates with IL-10. J Immunol (2004) 173(7):4479–91. 10.4049/jimmunol.173.7.4479 15383579

[199] Sweet RA Christensen SR Harris ML Shupe J Sutherland JL Shlomchik MJ A new site-directed transgenic rheumatoid factor mouse model demonstrates extrafollicular class switch and plasmablast formation. Autoimmunity (2010) 43(8):607–18. 10.3109/08916930903567500 20370572 PMC3818904

[200] Arce E Jackson DG Gill MA Bennett LB Banchereau J Pascual V. Increased frequency of pre-germinal center B cells and plasma cell precursors in the blood of children with systemic lupus erythematosus. J Immunol (2001) 167(4):2361–9. 10.4049/jimmunol.167.4.2361 11490026

[201] Zhang X Lindwall E Gauthier C Lyman J Spencer N Alarakhia A Circulating CXCR5+CD4+helper T cells in systemic lupus erythematosus patients share phenotypic properties with germinal center follicular helper T cells and promote antibody production. Lupus (2015) 24(9):909–17. 10.1177/0961203314567750 25654980

[202] Brink R. The imperfect control of self-reactive germinal center B cells. Curr Opin Immunol (2014) 28:97–101. 10.1016/j.coi.2014.03.001 24686094

[203] Guo W Smith D Aviszus K Detanico T Heiser RA Wysocki LJ. Somatic hypermutation as a generator of antinuclear antibodies in a murine model of systemic autoimmunity. J Exp Med (2010) 207(10):2225–37. 10.1084/jem.20092712 20805563 PMC2947070

[204] Mietzner B Tsuiji M Scheid J Velinzon K Tiller T Abraham K Autoreactive IgG memory antibodies in patients with systemic lupus erythematosus arise from nonreactive and polyreactive precursors. Proc Natl Acad Sci U S A (2008) 105(28):9727–32. 10.1073/pnas.0803644105 18621685 PMC2474524

[205] Paul E Nelde A Verschoor A Carroll MC. Follicular exclusion of autoreactive B cells requires FcgammaRIIb. Int Immunol (2007) 19(4):365–73. 10.1093/intimm/dxm002 17307801

[206] Dai X James RG Habib T Singh S Jackson S Khim S A disease-associated PTPN22 variant promotes systemic autoimmunity in murine models. J Clin Invest (2013) 123(5):2024–36. 10.1172/JCI66963 23619366 PMC3638909

[207] Nowosad CR Spillane KM Tolar P. Germinal center B cells recognize antigen through a specialized immune synapse architecture. Nat Immunol (2016) 17(7):870–7. 10.1038/ni.3458 27183103 PMC4943528

[208] Walsh ER Pisitkun P Voynova E Deane JA Scott BL Caspi RR Dual signaling by innate and adaptive immune receptors is required for TLR7-induced B-cell-mediated autoimmunity. Proc Natl Acad Sci U S A (2012) 109(40):16276–81. 10.1073/pnas.1209372109 22988104 PMC3479588

[209] Jackson SW Scharping NE Kolhatkar NS Khim S Schwartz MA Li QZ Opposing impact of B cell-intrinsic TLR7 and TLR9 signals on autoantibody repertoire and systemic inflammation. J Immunol (2014) 192(10):4525–32. 10.4049/jimmunol.1400098 24711620 PMC4041708

[210] Das A Heesters BA Bialas A O’Flynn J Rifkin IR Ochando J Follicular dendritic cell activation by TLR Ligands promotes autoreactive B cell responses. Immunity (2017) 46(1):106–19. 10.1016/j.immuni.2016.12.014 28099860 PMC8140609

[211] Woods M Zou YR Davidson A. Defects in germinal center selection in SLE. Front Immunol (2015) 6:425. 10.3389/fimmu.2015.00425 26322049 PMC4536402

[212] Diamond B Scharff MD. Somatic mutation of the T15 heavy chain gives rise to an antibody with autoantibody specificity. Proc Natl Acad Sci U S A (1984) 81(18):5841–4. 10.1073/pnas.81.18.5841 6435121 PMC391807

[213] Zhang J Roschke V Baker KP Wang Z Alarcon GS Fessler BJ Cutting edge: a role for B lymphocyte stimulator in systemic lupus erythematosus. J Immunol (2001) 166(1):6–10. 10.4049/jimmunol.166.1.6 11123269

[214] Coquery CM Loo WM Wade NS Bederman AG Tung KS Lewis JE BAFF regulates follicular helper t cells and affects their accumulation and interferon-gamma production in autoimmunity. Arthritis Rheumatol (2015) 67(3):773–84. 10.1002/art.38950 25385309 PMC4342294

[215] Mackay F Woodcock SA Lawton P Ambrose C Baetscher M Schneider P Mice transgenic for BAFF develop lymphocytic disorders along with autoimmune manifestations. J Exp Med (1999) 190(11):1697–710. 10.1084/jem.190.11.1697 10587360 PMC2195729

[216] Simpson N Gatenby PA Wilson A Malik S Fulcher DA Tangye SG Expansion of circulating T cells resembling follicular helper T cells is a fixed phenotype that identifies a subset of severe systemic lupus erythematosus. Arthritis Rheum (2010) 62(1):234–44. 10.1002/art.25032 20039395

[217] Nakou M Papadimitraki ED Fanouriakis A Bertsias GK Choulaki C Goulidaki N Interleukin-21 is increased in active systemic lupus erythematosus patients and contributes to the generation of plasma B cells. Clin Exp Rheumatol (2013) 31(2):172–9. 23137515

[218] Wong CK Wong PT Tam LS Li EK Chen DP Lam CW. Elevated production of B cell chemokine CXCL13 is correlated with systemic lupus erythematosus disease activity. J Clin Immunol (2010) 30(1):45–52. 10.1007/s10875-009-9325-5 19774453

[219] Linterman MA Rigby RJ Wong RK Yu D Brink R Cannons JL Follicular helper T cells are required for systemic autoimmunity. J Exp Med (2009) 206(3):561–76. 10.1084/jem.20081886 19221396 PMC2699132

[220] Jacquemin C Schmitt N Contin-Bordes C Liu Y Narayanan P Seneschal J OX40 ligand contributes to human lupus pathogenesis by promoting T follicular helper response. Immunity (2015) 42(6):1159–70. 10.1016/j.immuni.2015.05.012 26070486 PMC4570857

[221] Luo J Niu X Liu H Zhang M Chen M Deng S. Up-regulation of transcription factor Blimp1 in systemic lupus erythematosus. Mol Immunol (2013) 56(4):574–82. 10.1016/j.molimm.2013.05.241 23911415

[222] Guimaraes PM Scavuzzi BM Stadtlober NP Franchi Santos L Lozovoy MAB Iriyoda TMV Cytokines in systemic lupus erythematosus: far beyond Th1/Th2 dualism lupus: cytokine profiles. Immunol Cell Biol (2017) 95(9):824–31. 10.1038/icb.2017.53 28649995

[223] Salazar-Camarena DC Ortiz-Lazareno PC Cruz A Oregon-Romero E Machado-Contreras JR Munoz-Valle JF Association of BAFF, APRIL serum levels, BAFF-R, TACI and BCMA expression on peripheral B-cell subsets with clinical manifestations in systemic lupus erythematosus. Lupus (2016) 25(6):582–92. 10.1177/0961203315608254 26424128

[224] Hoyer BF Moser K Hauser AE Peddinghaus A Voigt C Eilat D Short-lived plasmablasts and long-lived plasma cells contribute to chronic humoral autoimmunity in NZB/W mice. J Exp Med (2004) 199(11):1577–84. 10.1084/jem.20040168 15173206 PMC2211779

[225] Lo MS Zurakowski D Son MB Sundel RP. Hypergammaglobulinemia in the pediatric population as a marker for underlying autoimmune disease: a retrospective cohort study. Pediatr Rheumatol Online J (2013) 11(1):42. 10.1186/1546-0096-11-42 24180594 PMC3831248

[226] Cassese G Lindenau S de Boer B Arce S Hauser A Riemekasten G Inflamed kidneys of NZB/W mice are a major site for the homeostasis of plasma cells. Eur J Immunol (2001) 31(9):2726–32. 10.1002/1521-4141(200109)31:9<2726::AID-IMMU2726>3.0.CO;2-H 11536171

[227] Starke C Frey S Wellmann U Urbonaviciute V Herrmann M Amann K High frequency of autoantibody-secreting cells and long-lived plasma cells within inflamed kidneys of NZB/W F1 lupus mice. Eur J Immunol (2011) 41(7):2107–12. 10.1002/eji.201041315 21484784

[228] Wang A Fairhurst AM Tus K Subramanian S Liu Y Lin F CXCR4/CXCL12 hyperexpression plays a pivotal role in the pathogenesis of lupus. J Immunol (2009) 182(7):4448–58. 10.4049/jimmunol.0801920 19299746 PMC2946082

[229] Deng Y Tsao BP. Updates in lupus genetics. Curr Rheumatol Rep (2017) 19(11):68. 10.1007/s11926-017-0695-z 28983873

[230] Vaughn SE Kottyan LC Munroe ME Harley JB. Genetic susceptibility to lupus: the biological basis of genetic risk found in B cell signaling pathways. J Leukoc Biol (2012) 92(3):577–91. 10.1189/jlb.0212095 22753952 PMC3748338

[231] Teruel M Alarcon-Riquelme ME. The genetic basis of systemic lupus erythematosus: what are the risk factors and what have we learned. J Autoimmun (2016) 74:161–75. 10.1016/j.jaut.2016.08.001 27522116

[232] Ghodke-Puranik Y Niewold TB. Immunogenetics of systemic lupus erythematosus: a comprehensive review. J Autoimmun (2015) 64:125–36. 10.1016/j.jaut.2015.08.004 26324017 PMC4628859

[233] Zhou XJ Cheng FJ Qi YY Zhao MH Zhang H A replication study from Chinese supports association between lupus-risk allele in TNFSF4 and renal disorder. Biomed Res Int (2013) 2013:597921 10.1155/2013/597921 23936824 PMC3713374

[234] Manku H Langefeld CD Guerra SG Malik TH Alarcon-Riquelme M Anaya JM Trans-ancestral studies fine map the SLE-susceptibility locus TNFSF4. PLoS Genet (2013) 9(7):e1003554. 10.1371/journal.pgen.1003554 23874208 PMC3715547

[235] Lamagna C Hu Y DeFranco AL Lowell CA. B cell-specific loss of Lyn kinase leads to autoimmunity. J Immunol (2014) 192(3):919–28. 10.4049/jimmunol.1301979 24376269 PMC3900234

[236] Hua Z Gross AJ Lamagna C Ramos-Hernandez N Scapini P Ji M Requirement for MyD88 signaling in B cells and dendritic cells for germinal center anti-nuclear antibody production in Lyn-deficient mice. J Immunol (2014) 192(3):875–85. 10.4049/jimmunol.1300683 24379120 PMC4101002

[237] Simpfendorfer KR Olsson LM Manjarrez Orduno N Khalili H Simeone AM Katz MS The autoimmunity-associated BLK haplotype exhibits cis-regulatory effects on mRNA and protein expression that are prominently observed in B cells early in development. Hum Mol Genet (2012) 21(17):3918–25. 10.1093/hmg/dds220 22678060 PMC3412385

[238] Simpfendorfer KR Armstead BE Shih A Li W Curran M Manjarrez-Orduno N Autoimmune disease-associated haplotypes of BLK exhibit lowered thresholds for B cell activation and expansion of Ig class-switched B cells. Arthritis Rheumatol (2015) 67(11):2866–76. 10.1002/art.39301 26246128

[239] Samuelson EM Laird RM Maue AC Rochford R Hayes SM. Blk haploin-sufficiency impairs the development, but enhances the functional responses, of MZ B cells. Immunol Cell Biol (2012) 90(6):620–9. 10.1038/icb.2011.76 21894171 PMC4096015

[240] Texido G Su IH Mecklenbrauker I Saijo K Malek SN Desiderio S The B-cell-specific Src-family kinase Blk is dispensable for B-cell development and activation. Mol Cell Biol (2000) 20(4):1227–33. 10.1128/MCB.20.4.1227-1233.2000 10648608 PMC85250

[241] Kozyrev SV Abelson AK Wojcik J Zaghlool A Linga Reddy MV Sanchez E Functional variants in the B-cell gene BANK1 are associated with systemic lupus erythematosus. Nat Genet (2008) 40(2):211–6. 10.1038/ng.79 18204447

[242] Yokoyama K Su Ih IH Tezuka T Yasuda T Mikoshiba K Tarakhovsky A BANK regulates BCR-induced calcium mobilization by promoting tyrosine phosphorylation of IP(3) receptor. EMBO J (2002) 21(1–2):83–92. 10.1093/emboj/21.1.83 11782428 PMC125810

[243] Aiba Y Yamazaki T Okada T Gotoh K Sanjo H Ogata M BANK negatively regulates Akt activation and subsequent B cell responses. Immunity (2006) 24(3):259–68. 10.1016/j.immuni.2006.01.002 16546095

[244] Rieck M Arechiga A Onengut-Gumuscu S Greenbaum C Concannon P Buckner JH. Genetic variation in PTPN22 corresponds to altered function of T and B lymphocytes. J Immunol (2007) 179(7):4704–10. 10.4049/jimmunol.179.7.4704 17878369

[245] Hasegawa K Martin F Huang G Tumas D Diehl L Chan AC PEST domain-enriched tyrosine phosphatase (PEP) regulation of effector/memory T cells. Science (2004) 303(5658):685–9. 10.1126/science.1092138 14752163

[246] Schickel JN Kuhny M Baldo A Bannock JM Massad C Wang H PTPN22 inhibition resets defective human central B cell tolerance. Sci Immunol (2016) 1(1):aaf7153 10.1126/sciimmunol.aaf7153 PMC5127630 27917411

[247] Vang T Congia M Macis MD Musumeci L Orru V Zavattari P Autoimmune-associated lymphoid tyrosine phosphatase is a gain-of-function variant. Nat Genet (2005) 37(12):1317–9. 10.1038/ng1673 16273109

[248] Graham RR Cotsapas C Davies L Hackett R Lessard CJ Leon JM Genetic variants near TNFAIP3 on 6q23 are associated with systemic lupus erythematosus. Nat Genet (2008) 40(9):1059–61. 10.1038/ng.200 19165918 PMC2772171

[249] Musone SL Taylor KE Lu TT Nititham J Ferreira RC Ortmann W Multiple polymorphisms in the TNFAIP3 region are independently associated with systemic lupus erythematosus. Nat Genet (2008) 40(9):1062–4. 10.1038/ng.202 19165919 PMC3897246

[250] Adrianto I Wen F Templeton A Wiley G King JB Lessard CJ Association of a functional variant downstream of TNFAIP3 with systemic lupus erythematosus. Nat Genet (2011) 43(3):253–8. 10.1038/ng.766 21336280 PMC3103780

[251] Tavares RM Turer EE Liu CL Advincula R Scapini P Rhee L The ubiquitin modifying enzyme A20 restricts B cell survival and prevents autoimmunity. Immunity (2010) 33(2):181–91. 10.1016/j.immuni.2010.07.017 20705491 PMC2931361

[252] Chu Y Vahl JC Kumar D Heger K Bertossi A Wojtowicz E B cells lacking the tumor suppressor TNFAIP3/A20 display impaired differentiation and hyperactivation and cause inflammation and autoimmunity in aged mice. Blood (2011) 117(7):2227–36. 10.1182/blood-2010-09-306019 21088135

[253] Hovelmeyer N Reissig S Xuan NT Adams-Quack P Lukas D Nikolaev A A20 deficiency in B cells enhances B-cell proliferation and results in the development of autoantibodies. Eur J Immunol (2011) 41(3):595–601. 10.1002/eji.201041313 21341261

[254] Adrianto I Wang S Wiley GB Lessard CJ Kelly JA Adler AJ Association of two independent functional risk haplotypes in TNIP1 with systemic lupus erythematosus. Arthritis Rheum (2012) 64(11):3695–705. 10.1002/art.34642 22833143 PMC3485412

[255] Nanda SK Venigalla RK Ordureau A Patterson-Kane JC Powell DW Toth R Polyubiquitin binding to ABIN1 is required to prevent autoimmunity. J Exp Med (2011) 208(6):1215–28. 10.1084/jem.20102177 21606507 PMC3173241

[256] Manjarrez-Orduno N Marasco E Chung SA Katz MS Kiridly JF Simpfendorfer KR CSK regulatory polymorphism is associated with systemic lupus erythematosus and influences B-cell signaling and activation. Nat Genet (2012) 44(11):1227–30. 10.1038/ng.2439 23042117 PMC3715052

[257] Floto RA Clatworthy MR Heilbronn KR Rosner DR MacAry PA Rankin A Loss of function of a lupus-associated FcgammaRIIb polymorphism through exclusion from lipid rafts. Nat Med (2005) 11(10):1056–8. 10.1038/nm1288 16170323

[258] Blank MC Stefanescu RN Masuda E Marti F King PD Redecha PB Decreased transcription of the human FCGR2B gene mediated by the -343 G/C promoter polymorphism and association with systemic lupus erythematosus. Hum Genet (2005) 117(2–3):220–7. 10.1007/s00439-005-1302-3 15895258

[259] Kono H Kyogoku C Suzuki T Tsuchiya N Honda H Yamamoto K FcgammaRIIB Ile232Thr transmembrane polymorphism associated with human systemic lupus erythematosus decreases affinity to lipid rafts and attenuates inhibitory effects on B cell receptor signaling. Hum Mol Genet (2005) 14(19):2881–92. 10.1093/hmg/ddi320 16115811

[260] Tiller T Kofer J Kreschel C Busse CE Riebel S Wickert S Development of self-reactive germinal center B cells and plasma cells in autoimmune Fc gammaRIIB-deficient mice. J Exp Med (2010) 207(12):2767–78. 10.1084/jem.20100171 21078890 PMC2989760

[261] Takai T Ono M Hikida M Ohmori H Ravetch JV. Augmented humoral and anaphylactic responses in Fc gamma RII-deficient mice. Nature (1996) 379(6563):346–9. 10.1038/379346a0 8552190

[262] Xiang Z Cutler AJ Brownlie RJ Fairfax K Lawlor KE Severinson E FcgammaRIIb controls bone marrow plasma cell persistence and apoptosis. Nat Immunol (2007) 8(4):419–29. 10.1038/ni1440 17322888

[263] Niewold TB Kelly JA Flesch MH Espinoza LR Harley JB Crow MK. Association of the IRF5 risk haplotype with high serum interferon-alpha activity in systemic lupus erythematosus patients. Arthritis Rheum (2008) 58(8):2481–7. 10.1002/art.23613 18668568 PMC2621107

[264] Feng D Stone RC Eloranta ML Sangster-Guity N Nordmark G Sigurdsson S Genetic variants and disease-associated factors contribute to enhanced interferon regulatory factor 5 expression in blood cells of patients with systemic lupus erythematosus. Arthritis Rheum (2010) 62(2):562–73. 10.1002/art.27223 20112383 PMC3213692

[265] Purtha WE Swiecki M Colonna M Diamond MS Bhattacharya D. Spontaneous mutation of the Dock2 gene in Irf5-/- mice complicates interpretation of type I interferon production and antibody responses. Proc Natl Acad Sci U S A (2012) 109(15):E898–904. 10.1073/pnas.1118155109 22431588 PMC3326475

[266] Yasuda K Watkins AA Kochar GS Wilson GE Laskow B Richez C Interferon regulatory factor-5 deficiency ameliorates disease severity in the MRL/lpr mouse model of lupus in the absence of a mutation in DOCK2. PLoS One (2014) 9(7):e103478. 10.1371/journal.pone.0103478 25076492 PMC4116215

[267] Thackray LB Shrestha B Richner JM Miner JJ Pinto AK Lazear HM Interferon regulatory factor 5-dependent immune responses in the draining lymph node protect against West Nile virus infection. J Virol (2014) 88(19):11007–21. 10.1128/JVI.01545-14 25031348 PMC4178807

[268] Abelson AK Delgado-Vega AM Kozyrev SV Sanchez E Velazquez-Cruz R Eriksson N STAT4 associates with systemic lupus erythematosus through two independent effects that correlate with gene expression and act additively with IRF5 to increase risk. Ann Rheum Dis (2009) 68(11):1746–53. 10.1136/ard.2008.097642 19019891 PMC3878433

[269] Liang Y Pan HF Ye DQ. Therapeutic potential of STAT4 in autoimmunity. Expert Opin Ther Targets (2014) 18(8):945–60. 10.1517/14728222.2014.920325 24844303

[270] Xu Z Duan B Croker BP Morel L. STAT4 deficiency reduces autoantibody production and glomerulonephritis in a mouse model of lupus. Clin Immunol (2006) 120(2):189–98. 10.1016/j.clim.2006.03.009 16713741

[271] Muto A Ochiai K Kimura Y Itoh-Nakadai A Calame KL Ikebe D Bach2 represses plasma cell gene regulatory network in B cells to promote antibody class switch. EMBO J (2010) 29(23):4048–61. 10.1038/emboj.2010.257 20953163 PMC3020649

[272] Morris DL Sheng Y Zhang Y Wang YF Zhu Z Tombleson P Genome-wide association meta-analysis in Chinese and European individuals identifies ten new loci associated with systemic lupus erythematosus. Nat Genet (2016) 48(8):940–6. 10.1038/ng.3603 27399966 PMC4966635

[273] Hipp N Symington H Pastoret C Caron G Monvoisin C Tarte K IL-2 imprints human naive B cell fate towards plasma cell through ERK/ELK1-mediated BACH2 repression. Nat Commun (2017) 8(1):1443. 10.1038/s41467-017-01475-7 29129929 PMC5682283

[274] Kim SJ Gregersen PK Diamond B. Regulation of dendritic cell activation by microRNA let-7c and BLIMP1. J Clin Invest (2013) 123(2):823–33. 10.1172/JCI64712 23298838 PMC3561813

[275] Kim SJ Schatzle S Ahmed SS Haap W Jang SH Gregersen PK Increased cathepsin S in Prdm1-/- dendritic cells alters the TFH cell repertoire and contributes to lupus. Nat Immunol (2017) 18(9):1016–24. 10.1038/ni.3793 28692065 PMC5568473

[276] Cunninghame Graham DS Morris DL Bhangale TR Criswell LA Syvanen AC Ronnblom L Association of NCF2, IKZF1, IRF8, IFIH1, and TYK2 with systemic lupus erythematosus. PLoS Genet (2011) 7(10):e1002341 10.1371/journal.pgen.1002341 22046141 PMC3203198

[277] Carotta S Willis SN Hasbold J Inouye M Pang SH Emslie D The transcription factors IRF8 and PU.1 negatively regulate plasma cell differentiation. J Exp Med (2014) 211(11):2169–81. 10.1084/jem.20140425 25288399 PMC4203955

[278] Lee CH Melchers M Wang H Torrey TA Slota R Qi CF Regulation of the germinal center gene program by interferon (IFN) regulatory factor 8/IFN consensus sequence-binding protein. J Exp Med (2006) 203(1):63–72. 10.1084/jem.20051450 16380510 PMC2118063

[279] Cai X Huang W Liu X Wang L Jiang Y. Association of novel polymorphisms in TMEM39A gene with systemic lupus erythematosus in a Chinese Han population. BMC Med Genet (2017) 18(1):43. 10.1186/s12881-017-0405-8 28427360 PMC5399404

[280] Cortes M Georgopoulos K. Aiolos is required for the generation of high affinity bone marrow plasma cells responsible for long-term immunity. J Exp Med (2004) 199(2):209–19. 10.1084/jem.20031571 14718515 PMC2211773

[281] Wang JH Avitahl N Cariappa A Friedrich C Ikeda T Renold A Aiolos regulates B cell activation and maturation to effector state. Immunity (1998) 9(4):543–53. 10.1016/S1074-7613(00)80637-8 9806640

[282] Russell L John S Cullen J Luo W Shlomchik MJ Garrett-Sinha LA. Requirement for transcription factor Ets1 in B cell tolerance to self-antigens. J Immunol (2015) 195(8):3574–83. 10.4049/jimmunol.1500776 26355157 PMC4568556

[283] John SA Clements JL Russell LM Garrett-Sinha LA. Ets-1 regulates plasma cell differentiation by interfering with the activity of the transcription factor Blimp-1. J Biol Chem (2008) 283(2):951–62. 10.1074/jbc.M705262200 17977828

[284] Lu X Zoller EE Weirauch MT Wu Z Namjou B Williams AH Lupus risk variant increases pSTAT1 binding and decreases ETS1 expression. Am J Hum Genet (2015) 96(5):731–9. 10.1016/j.ajhg.2015.03.002 25865496 PMC4570281

[285] Yang W Shen N Ye DQ Liu Q Zhang Y Qian XX Genome-wide association study in Asian populations identifies variants in ETS1 and WDFY4 associated with systemic lupus erythematosus. PLoS Genet (2010) 6(2):e1000841. 10.1371/journal.pgen.1000841 20169177 PMC2820522

[286] Ols ML Cullen JL Turqueti-Neves A Giles J Shlomchik MJ. Dendritic cells regulate extrafollicular autoreactive B cells via T cells expressing Fas and Fas ligand. Immunity (2016) 45(5):1052–65. 10.1016/j.immuni.2016.10.005 27793595 PMC5112117

[287] Wu J Metz C Xu X Abe R Gibson AW Edberg JC A novel polymorphic CAAT/enhancer-binding protein beta element in the FasL gene promoter alters Fas ligand expression: a candidate background gene in African American systemic lupus erythematosus patients. J Immunol (2003) 170(1):132–8. 10.4049/jimmunol.170.1.132 12496392

[288] Shen N Fu Q Deng Y Qian X Zhao J Kaufman KM Sex-specific association of X-linked toll-like receptor 7 (TLR7) with male systemic lupus erythematosus. Proc Natl Acad Sci U S A (2010) 107(36):15838–43. 10.1073/pnas.1001337107 20733074 PMC2936646

[289] Soni C Wong EB Domeier PP Khan TN Satoh T Akira S B cell-intrinsic TLR7 signaling is essential for the development of spontaneous germinal centers. J Immunol (2014) 193(9):4400–14. 10.4049/jimmunol.1401720 25252960 PMC4201954

[290] Blasius AL Arnold CN Georgel P Rutschmann S Xia Y Lin P Slc15a4, AP-3, and Hermansky-Pudlak syndrome proteins are required for toll-like receptor signaling in plasmacytoid dendritic cells. Proc Natl Acad Sci U S A (2010) 107(46):19973–8. 10.1073/pnas.1014051107 21045126 PMC2993408

[291] Sasawatari S Okamura T Kasumi E Tanaka-Furuyama K Yanobu-Takanashi R Shirasawa S The solute carrier family 15A4 regulates TLR9 and NOD1 functions in the innate immune system and promotes colitis in mice. Gastroenterology (2011) 140(5):1513–25. 10.1053/j.gastro.2011.01.041 21277849

[292] Kobayashi T Shimabukuro-Demoto S Yoshida-Sugitani R Furuyama-Tanaka K Karyu H Sugiura Y The histidine transporter SLC15A4 coordinates mTOR-dependent inflammatory responses and pathogenic antibody production. Immunity (2014) 41(3):375–88. 10.1016/j.immuni.2014.08.011 25238095

[293] Baccala R Gonzalez-Quintial R Blasius AL Rimann I Ozato K Kono DH Essential requirement for IRF8 and SLC15A4 implicates plasmacytoid dendritic cells in the pathogenesis of lupus. Proc Natl Acad Sci U S A (2013) 110(8):2940–5. 10.1073/pnas.1222798110 23382217 PMC3581947

